# Interactive cognitive maps support flexible behavior under threat

**DOI:** 10.1016/j.celrep.2023.113008

**Published:** 2023-08-22

**Authors:** Toby Wise, Caroline J. Charpentier, Peter Dayan, Dean Mobbs

**Affiliations:** 1 Department of Neuroimaging, Institute of Psychiatry, Psychology & Neuroscience, King’s College London, London, UK; 2 Department of Humanities and Social Sciences, California Institute of Technology, Pasadena, CA, USA; 3 Department of Psychology, University of Maryland, College Park, MD, USA; 4 Brain and Behavior Institute, University of Maryland, College Park, MD, USA; 5 Max Planck Institute for Biological Cybernetics, Tübingen, Germany; 6 University of Tübingen, Tübingen, Germany; 7 Computation and Neural Systems Program, California Institute of Technology, Pasadena, CA, USA; 8 Lead contact

## Abstract

In social environments, survival can depend upon inferring and adapting to other agents’ goal-directed behavior. However, it remains unclear how humans achieve this, despite the fact that many decisions must account for complex, dynamic agents acting according to their own goals. Here, we use a predator-prey task (total n=510) to demonstrate that humans exploit an interactive cognitive map of the social environment to infer other agents’ preferences and simulate their future behavior, providing for flexible, generalizable responses. A model-based inverse reinforcement learning model explained participants’ inferences about threatening agents’ preferences, with participants using this inferred knowledge to enact generalizable, model-based behavioral responses. Using tree-search planning models, we then found that behavior was best explained by a planning algorithm that incorporated simulations of the threat’s goal-directed behavior. Our results indicate that humans use a cognitive map to determine other agents’ preferences, facilitating generalized predictions of their behavior and effective responses.

## In brief

Wise et al. characterize the computational mechanisms that enable humans to gain reward and avoid danger in the presence of other agents, demonstrating that these abilities rely on the ability to exploit an internal model of the environment that incorporates predictions about others’ goals and actions.

## Graphical abstract

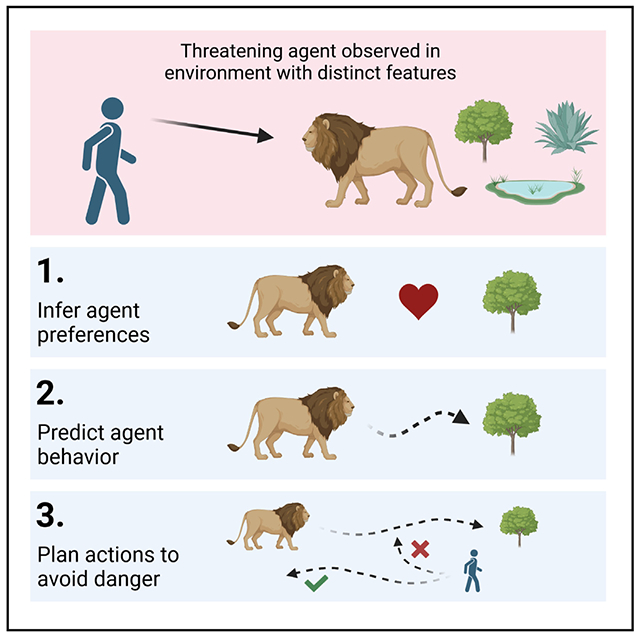

## INTRODUCTION

Our ability to predict and adapt to others’ behavior is one that we use regularly and often seemingly automatically.^1,3^ One salient example of this behavior is threat avoidance; in the natural world, organisms proactively infer predators’ goals and predict their movements so as to make better avoidance decisions.^2^ Rudimentary aspects of these abilities are observed across species, where animals will learn the behaviors of their predators and act according to this information to avoid predation.^4^ Yet, humans are particularly astute at inferring mental states and predicting behaviors of complex agents,^5^ whether threatening or not, an ability that is likely critical in modern society, where many everyday actions depend upon interactions with other humans. Despite the enormous survival advantage of these abilities, the complex computations that enable us to simulate a threat’s goal-directed locomotion and respond appropriately remain poorly understood. Here, we show that humans’ ability to avoid dynamic threats depends upon an internal model of the shared environment.

More generally, the ability to infer others’ goals and respond to their actions is well established.^1,3^ However, at a computational level it is a complex undertaking, and achieving humanlevel action prediction remains a significant challenge for artificial intelligence.^6^ Multiple systems are likely involved; while humans are adept at inferring others’ goals and predicting their resulting actions,^5^ it has also been shown that human participants adaptively switch between goal inference and computationally simpler strategies during observational learning according to the expected success of each strategy,^7^ suggesting that both approaches may be used in different situations depending on which performs best. Computational modeling has further revealed that human goal inference is supported by model-based planning processes^8^ (i.e., planning that uses explicit consideration of long-term outcomes based on an internal model of the world, as opposed to relying on habitual trialand-error learning), indicating that this process relies upon an internal model of the social environment. In addition to predicting others’ behavior, humans are also able to flexibly adapt their own behavior to account for these predictions. Computational modeling of simple social games has demonstrated that relatively complex decisions that account for others’ behavior can be recapitulated by planning algorithms,^9–11^ suggesting that planning processes can be adapted to incorporate predictions about other agents’ behavior as well as our own.^12,13^ Together, this work indicates that, while this type of behavior is automatic and intuitive,^3^ it is underpinned by complex computational mechanisms and relies on an accurate internal model of the social environment.

Predator-prey interactions provide an especially acute test of our ability to predict other agents’ actions given that suboptimal predictions may have immediately deleterious consequences. However, little is known about how the complex computational mechanisms that enable us to understand and predict social agents support avoidance. Avoidance tasks typically involve either static threats (e.g., place aversion) or simple mobile predators. In contrast, real-world environments feature dynamic social agents with their own goals and behavioral strategies, where these social prediction mechanisms will naturally assume greater importance. In addition, research on the computational underpinnings of social inference have typically used highly artificial tasks or games, and it is unclear how these mechanisms are deployed in naturalistic environments that better reflect the complexity of the real world.

**Figure 1.**
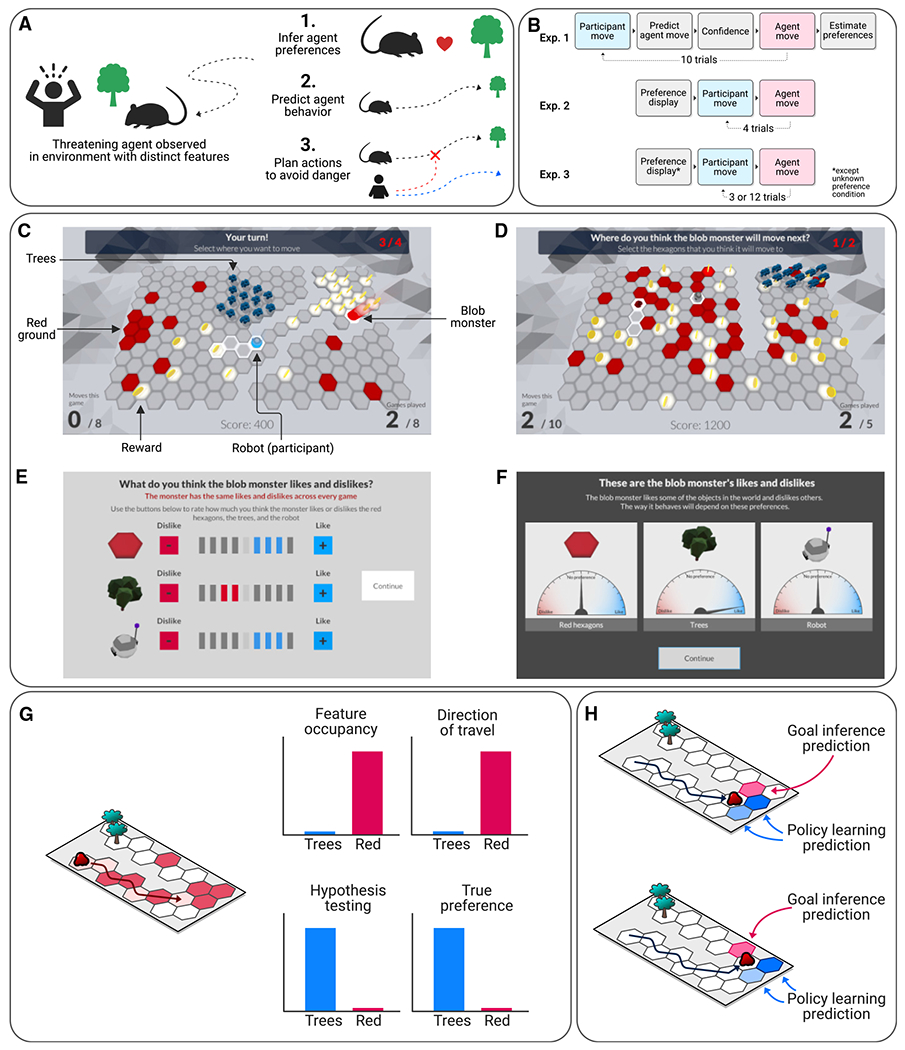
Overview and task design (A) Overview of proposed avoidance model. When faced with danger posed by a threatening agent, a human actor first infers the agent’s preferences before using these to predict its behavior and plan actions that will avoid an encounter with the threat. (B) Task timeline, indicating the different stages of the task across the three experiments. (C) Illustrative screenshot from the task. Participants controlled a robot exploring an environment containing two features (red ground and trees), alongside a blob monster that would eat the robot if the two occupied the same cell on the hexagonal grid. (D) In experiment 1, participants were asked to predict the threatening agent’s moves by selecting cells that they expected the agent to move to. (E) To assess how participants learned about the agent’s preferences, in experiment 1 they were asked to rate the agent’s likes and dislikes using a 9-point scale at the end of each game. (F) In other conditions (experiment 2, experiment 3, predictable and unpredictable conditions), participants were informed of the agent’s preferences prior to the game starting. (G) Illustration of how environments decoupled preferences from basic elements of behavior. Here, the monster has a preference for the trees and moves accordingly, but due to the environment layout it occupies red cells and travels primarily in the direction of red cells, while heading away from the trees. (H) Illustration of how this decoupling allowed goal inference and policy learning to be distinguished. Policy learning models predict that the agent will continue repeating the same moves it had made previously, moving either right or down. Goal inference models instead account for the fact that the agent will choose the action that brings it closest to the trees, which are its true preference.

Here, we seek to answer both of these questions. We use computational modeling to uncover the computational mechanisms that enable humans to avoid dynamic threats in complex virtual environments. While on the surface such successful avoidance behavior may not appear complex or noteworthy, our results indicate that this ability depends on sophisticated computational mechanisms. Together, our results indicate that humans infer predators’ goals by exploiting an internal model of the world. This inferred knowledge is then used to predict and simulate predators’ actions when planning, enabling flexible avoidance (Figure 1A). These findings outline a flexible process of social inference and decision-making, which may be uniquely human, that supports generalized avoidance.

## RESULTS

### Participants learn to predict threatening virtual agents’ actions

Five hundred and ten participants completed a task that involved moving in a 3D-rendered 2D virtual environment made up of hexagonal cells in order to collect rewards (represented by coins) while avoiding being eaten by a virtual threatening agent, described as a “blob monster” (Figures 1B–1F). Critically, the behavior of the threatening agent was guided by its specific preference (i.e., a non-zero reward weight) for only one of the three features of the environment (blue trees, red ground, or the gray “prey” robot, highlighted in cyan). This meant it would head toward the feature for which it had a preference. Participants played a number of games, each of which took place in a different environment, but with the predator’s preference remaining constant. Participants were instructed that the predator’s behavior would be guided by its preference for one of the three features.

In experiment 1 (n=150), our first question was whether participants could successfully learn another agent’s policy, which we tested by asking the participants to predict the action they expected the agent to take in a given state, accompanied by ratings of confidence in their predictions. They could accomplish this either by goal inference or by policy learning. On each trial, participants were asked to predict where they thought the agent would move prior to observing its actual movements. Importantly, the agent behaved predictably, with no stochasticity in its action selection, meaning that accurate prediction was possible if its preferences were learned accurately. This ensured that learning was not unnecessarily challenging for participants; while the complexity of the task meant that learning took time, successful learning was a prerequisite for evaluating models of *how* this learning occurred.

**Figure 2.**
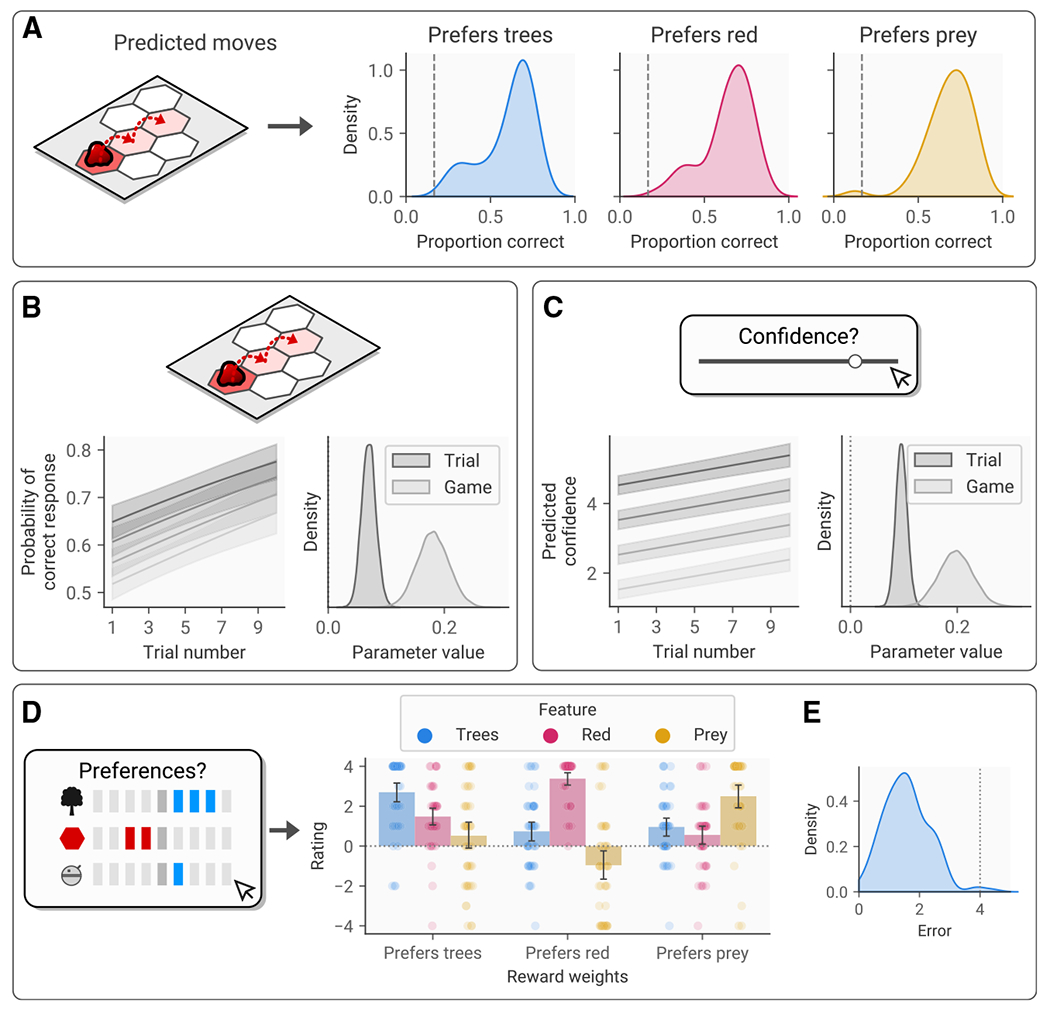
Participants’ predictions of threatening agent’s behavior and preferences (A) Proportion of threatening agent’s moves predicted correctly. Density plots represent the distribution of accuracy scores within the sample, calculated by taking the proportion of accurate predictions across all trials for each participant. Reward weight conditions correspond to the agent’s preferences for the trees, the red ground, or the robot prey. The dotted line represents the proportion of correct responses expected if selecting randomly. (B) Left: group-level posterior predicted probability of being correct from a hierarchical Bayesian regression model, demonstrating the effects of trial and game number on accuracy. Darker colors represent later games. Right: posterior distribution for the trial and game effect parameters in the prediction accuracy model. (C) Left: group-level posterior predicted confidence in action predictions from a hierarchical Bayesian regression model, demonstrating the effects of trial and game number on accuracy. Darker colors represent later games. Right: posterior distribution for the trial and game effect parameters in the confidence model. (D) Reported reward weights for the three conditions. Bars represent mean (±95% confidence interval), while points represent individual participants. (E) Prediction error across participants. The dotted line represents the expected error if participants were not learning the agent’s reward weights.

Across all three reward weight conditions in experiment 1, participants’ one-step predictions were significantly above chance (condition A, $t(49)=20.21,p=2.04\times 10^{-25},d=5.72$; condition $B,t(49)=23.09,p=5.15\times 10^{-28},d=6.53$; condition $C,t(49)=27.12,p=3.64\times 10^{-31},d=7.67$; Figure 2A), indicating that they were able to predict the agent’s actions accurately. Accuracy tended to improve across trials and games, as indicated by a Bayesian regression model predicting the probability of being correct from trial number (mean β=0.07,95% highest posterior density interval [HPDI]=[0.05,0.10]; Figure 2B) and game number (mean β=0.18,95%HPDI=[0.13,0.24]; Figure 2B), confirming that participants were learning about the agent’s policy incrementally as they completed the task. This was supported by an increase in confidence ratings across both trials (mean β=0.10,95%HPDI=[0.08,0.12]; Figure 2C) and games (mean β=0.20,95%HPDI=[0.14,0.26]; Figure 2C).

### Participants generalize across environments by learning threatening virtual agents’ reward weights

To understand whether participants had indeed learned the agent’s preferences from the observations in order to predict their actions, we collected participants’ estimates of the agent’s preferences for the three features in the environment (blue trees, red ground, and the robot character controlled by the participant) at the end of each game. Across all three reward weight conditions, participants were able to report the agent’s preferred feature significantly more accurately than would be expected if they had not learned its preferences (condition A, $t\left(49\right)=21.96,\text{p}=5.17\times 10^{-27},d=6.21$; condition $B,t\left(49\right)=27.62,p=1.57\times 10^{-31},d=7.81$; condition $C,t(49)=20.35,p=1.52\times 10^{-25},d=5.75$; Figure 2D). This effect was remarkably consistent across participants, with 99.3% of participants producing more accurate ratings than expected under this null hypothesis (Figure 2E).

### Action prediction is explained by a combination of policy learning and goal inference

Given participants’ ability to infer the agent’s reward function accurately, we expected that participants would use this information to inform their predictions of the agent’s actions. As such, we expected that behavior would be better explained by a model incorporating goal inference (i.e., predicting an agent’s action based on its reward function) than one using policy learning (i.e., learning the agent’s preferred action, independent of its goal alone). Critically, environments were designed such that the predator’s behavior was not trivially linked to the features of the environment (Figure 1G).

**Figure 3.**
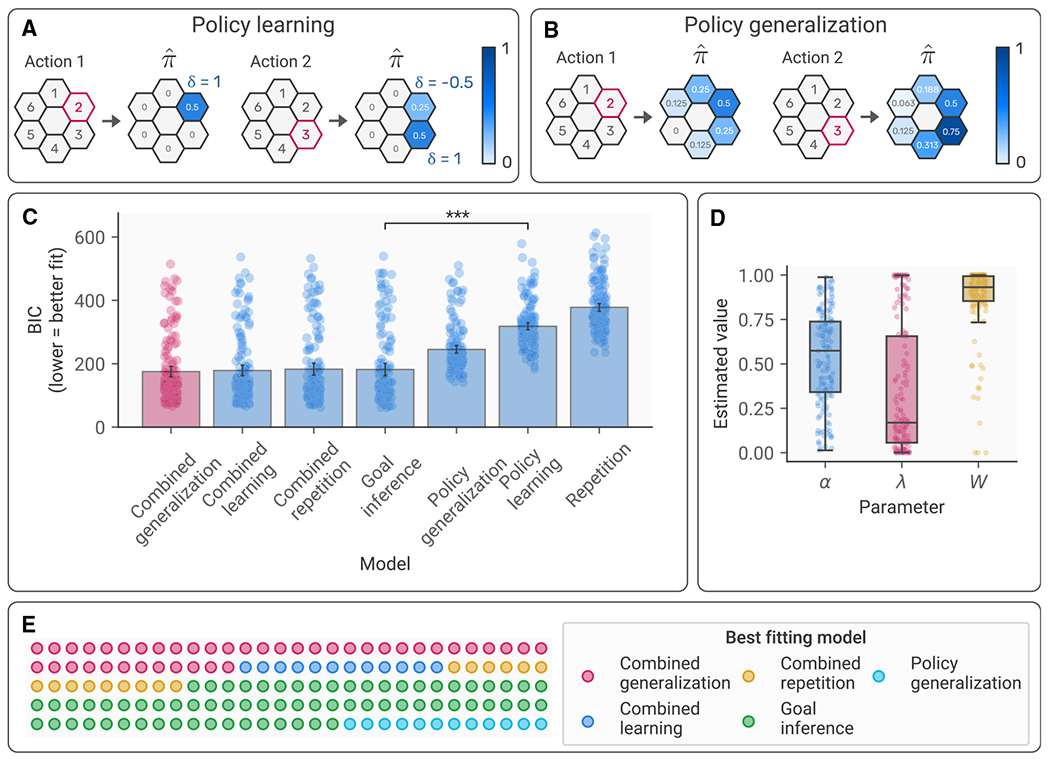
Computational modeling of action prediction (A) Illustration of the model-free policy learning model. Selected actions (highlighted in red) are imbued with a “value” that is dependent on the difference between the observed and the predicted action, weighted by a learning rate (here set to 0.5). The result is an inferred policy preference $\left(\hat{\pi }\right)$ for the agent. (B) Illustration of the model-free policy generalization model. This functions similar to the policy learning model, but the value of the action is generalized to adjacent actions according to a squared exponential kernel. (C) Model fit statistics (BIC) for each of the action prediction models tested. Bars represent the mean (±95% confidence interval) across participants, while points represent BIC values for individual participants. The model highlighted in red is the model with the lowest mean BIC, and the significance indicators correspond to the two *a priori* hypotheses tests designed to compare the combined model against the goal inference and policy learning models. ${}^{***}p<.001$. (D) Distribution of parameter values for the combined generalization model: α and λ represent the learning rate and learning rate decay, respectively, for the policy learning component, while W represents the contribution of goal inference in the combined model. Bars represent ±95% confidence intervals, while points represent individual subjects’ parameter estimates. (E) Best-fitting model for each subject in the sample.

We tested this using a series of computational models. The simplest policy learning model predicted the agent would repeat its previous action, ignoring its prior history. The next model learned a recency-weighted estimate of each action’s value (Figure 3A), while the final model generalized this learning process using a Gaussian kernel (Figure 3B), such that the value of states adjacent to the one chosen was also updated on each trial. The goal inference model, on the other hand, represented the task as a Markov decision problem (see STAR Methods) and determined the optimal policy for the Markov decision process (MDP) according to the agent’s true reward function. The agent’s next action was then predicted according to the resulting action values. Note that, while we refer to this as “goal inference,” as it predicts actions based on knowledge of the agent’s goals, for convenience we provide the model with the objective reward weights rather than requiring it to infer these. Random-effects analysis of model fit using Bayesian information criterion (BIC) scores supported our primary hypothesis, showing that the goal inference model fitted the data significantly better than the policy learning model $\left(t(149)=22.25,p=3.23\times 10^{-49},d=3.63\right.$; Figure 3C).

However, prior work has suggested that human participants rely on a combination of complex goal inference based on known preferences and simple policy learning.^14^ To test this, we performed exploratory analyses evaluating models that combined the predictions of the goal inference and policy learning models, weighting the predictions of each model according to an estimated weighting parameter W. Notably, model comparison indicated that a combination of goal inference and policy learning with generalization provided the best fit to the data of all the models tested (Figure 3C), suggesting that participants combined both strategies to an extent. However, estimated W parameter values indicated that participants tended to rely more heavily on goal inference (mean =0.87,SD=0.2; higher values indicate greater use of goal inference; Figure 3D), and goal inference alone was the most common best-fitting model across subjects (Figure 3E).

### Reward weight inference is best explained by a hypothesis-testing inverse reinforcement learning model

What are the computational mechanisms that enable participants to infer the threatening agent’s reward weights? To answer this question, we adopted a computational modeling framework based on inverse reinforcement learning,^6^ in which algorithms aim to learn an agent’s policy or reward weights based on observations of its actions.

**Figure 4.**
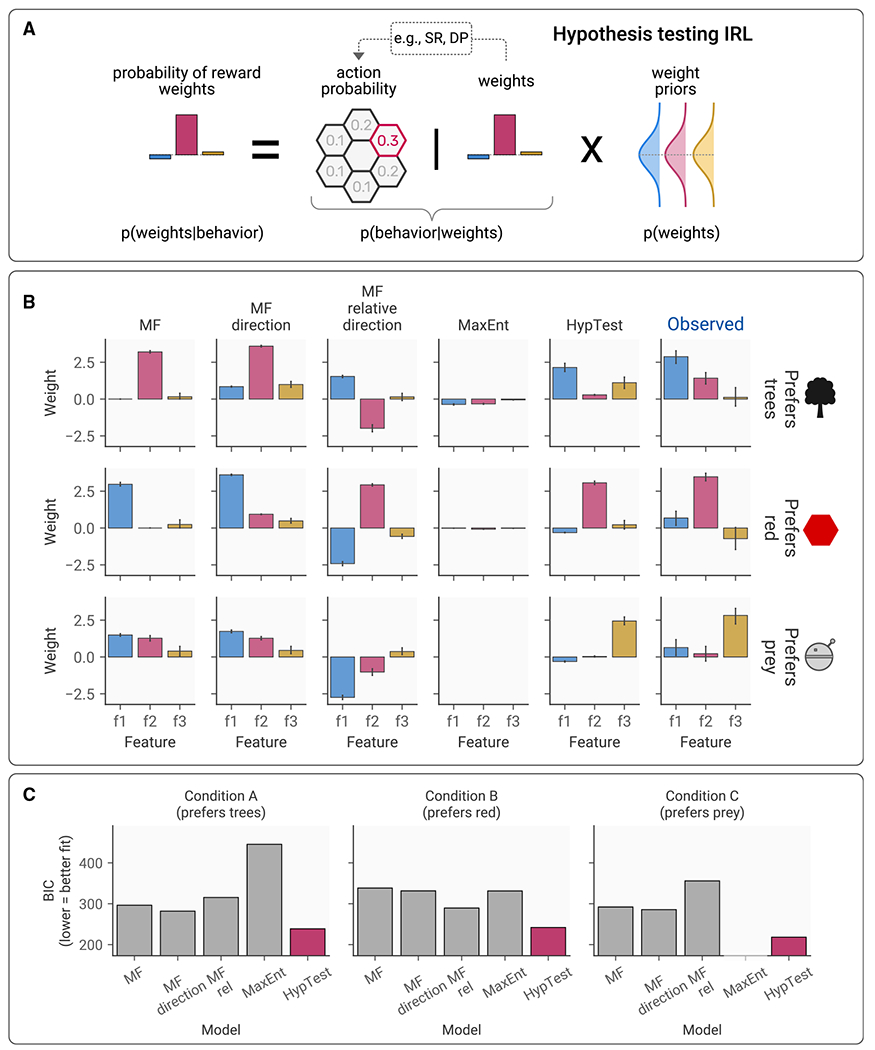
Computational modeling of preference ratings (A) Illustration of the hypothesis-testing inverse reinforcement learning (IRL) model, which uses Bayesian inference to determine the threatening agent’s reward weights based on predictions of its behavior generated according to candidate weights. The predictions can be provided by any valid method for determining action probabilities given a known reward function, such as successor representation (SR) or dynamic programming (DP). (B) Inverse reinforcement model predictions of agents’ reward weights, with participants’ predictions shown in the far right column. Note that the presence of error bars is inconsistent, as for some conditions all participants experienced the same agent, resulting in the same model predictions, while in others the agent’s actions differed across participants. Error bars represent ±95% confidence intervals. (C) Model fit statistics for the inverse reinforcement learning models tested, showing the BIC for each model across all predictions, where lower scores indicate better fit.

We developed a model inspired by work on hypothesis testing in human decision-making,^15^ alongside work on Bayesian inverse planning,^16^ which uses sampling-based Bayesian inference to predict the agent’s reward weights based on its behavior (referred to as HypTest; Figure 4A; see STAR Methods for details).

We compared our hypothesis-testing model against a selection of model-free inverse reinforcement learning algorithms (Figure 3A), which inferred the reward weights of the threatening agent based on the features it encountered or the features it was expected to encounter based on its direction of travel. This was necessary to confirm that observed behavior could not be explained by simpler approaches that did not rely on any form of task model. For completeness, we also tested an existing model-based inverse reinforcement learning algorithm from the maximum entropy family (MaxEnt).^19–21^ This feature-expectancy-based approach is commonly used within inverse reinforcement learning algorithms and has been successfully employed across a range of applications.^6^ As such, we included this as a useful comparison model that is known to be effective in many situations and seeks to learn reward weights rather than imitating a policy directly. While this model is similar to HypTest in that it incorporates knowledge of the task structure, MaxEnt assumes that the features in each state are stable throughout the agent’s trajectory. In condition 3, where the agent has a preference for the prey, the presence of the prey feature changes according to the prey’s movements, creating a situation in which MaxEnt cannot succeed (see STAR Methods for more detail).

Model comparison indicated that the hypothesis-testing model was able to recover the agent’s reward weights substantially more accurately than MaxEnt or model-free methods (Figure 4B), as shown across both adjusted $R^{2}$ (HypTest =0.09, next best model = −0.34) and BIC (HypTest =232.89, next best model =292.31; Figure 4C), suggesting that this model-based approach provides the best approximation of participants’ responses.

### Participants account for threatening agents’ goal-directed behavior during planning

**Figure 5.**
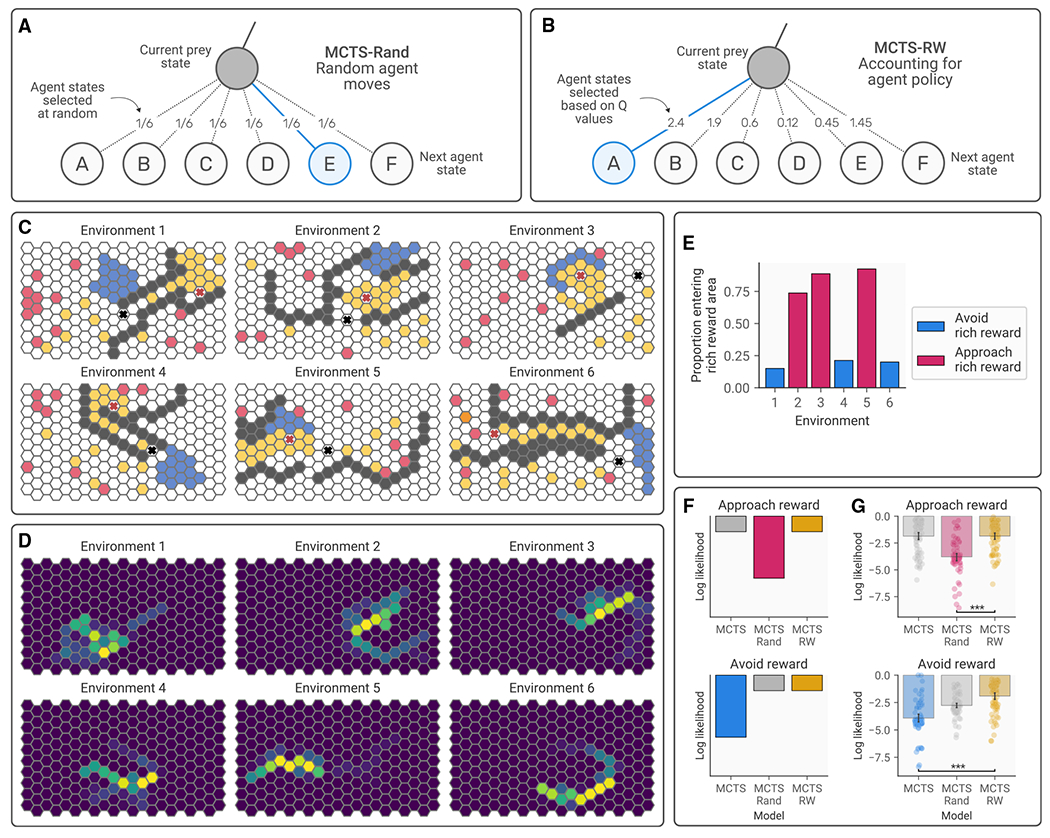
Computational modeling of participants’ action selection (A) Illustration of MCTS simulations, where the agent is assumed to move randomly (MCTS-Rand). (B) MCTS variant where the agent’s policy, based on known reward weights, is accounted for in the planning process (MCTS-RW). (C) Environments used in experiment 2. Yellow, reward; blue, trees; red, red ground; black, wall. The red and black crosses represent the agent and prey, respectively. In environments 1, 4, and 6, entering the area with concentrated rewards (the rich reward zone) will result in an encounter with the agent and should thus be avoided. In environments 2, 3, and 5, the rich reward zone can safely be entered as the agent will move toward the trees. (D) Heatmaps showing state occupancy across participants, with brighter colors representing states that are more frequently occupied. (E) Proportion of participants entering the rich reward zone in each environment, demonstrating that participants tend to enter when it is best to approach the rich rewards, but not when it is best to avoid. (F) Hypothesized results of planning model fitting. The top represents environments where the rich reward zone should be approached, where the critical comparison is between the MCTS variant that assumes the agent acts randomly (MCTS-Rand) and the MCTS variant that accounts for the agent’s goal-directed behavior based on its known reward weights (MCTS-RW). The bottom represents environments where the rich reward zone should be avoided, where the critical comparison is between non-interactive MCTS (which plans as if the agent did not exist) and MCTS-RW. (G) Results of model comparison, showing the log likelihood of each model for each participant, summed across environments within each condition. Error bars represent 95% confidence intervals, and the points represent individual participant log likelihoods. ${}^{**}\text{p}<.001$.

Given the ability of participants to infer the other agent’s reward weights, we reasoned that this should allow them to adapt their own plans depending on expectations of the agent’s goal-directed behavior. In experiment 2(n=80), we adapted the agent-prey task by creating environments that were carefully designed to test this hypothesis. Participants were presented with environments in which they could opt to move toward either a cluster of rewards or an area with sparser rewards. Critically, in some environments, heading toward the richer rewards would lead them into the path of the agent as it moved toward its goal, while in others the agent’s trajectory toward its goal would result in it avoiding the richly rewarded area or passing through the area rapidly. Participants were informed that the agent’s preference was just for the trees; although it would nevertheless eat the robot if their paths happened to cross. Thus, participants had no ambiguity about the preferences of the agent. They also made only four moves per turn, while the agent made six. Accordingly, if participants were accounting for the agent’s goal-directed planning based on known preferences, they should either head toward or away from the richly rewarded area, depending on whether they were likely to encounter the agent. In contrast, if they were not accounting for the agent at all, they should always head for the rich rewards. As expected, results demonstrated that participants typically headed for the sparsely rewarded regions when this would lead away from the path of the agent (environments 1, 2, and 5) and headed for the richly rewarded region when the agent’s trajectory enabled them to be avoided (environments 3, 4, and 6). A chi-squared test confirmed that a higher proportion of participants entered the richly rewarded zone when it was safe $\left(\chi^{2}(2)=131.44,\text{p}=\right.1.97\times 10^{-30}$; Figure 5E).

To provide further evidence for participants’ ability to plan interactively (i.e., accounting for the other agent’s behavior in their decision-making) and determine the computational mechanisms supporting this ability, we developed a series of planning models and fit these to participants’ behavior. The addition of another agent transforms the environment into an a more complex MDP, in which each state can be additionally defined by the position of the agent. This results in a large 210^2^ states based on predator and prey locations) MDP that is not straight-forwardly amenable to dynamic programming solutions. Instead, we turn to Monte Carlo tree search (MCTS) with a uniform rollout policy as a tree-search-based approximation method. To allow interactive planning, we augment the standard MCTS apparatus with knowledge about the agent’s expected actions to allow informed predictions about the consequences of the prey’s actions. We used two variants of this model, one that predicted the agent’s actions based on its known preference (MCTS-RW; Figure 5B) and one that assumed the agent chose its actions randomly (MCTS-Rand; Figure 5A). We compared these models to an MCTS model that ignored the presence of the agent entirely (MCTS).

In environments where it is safe to enter the rich reward area, MCTS-RW and MCTS predict that the participant will move toward the rich rewards, while MCTS-Rand avoids the rich rewards as it assumes the threatening agent may stray into this area (Figure 5F). In environments where it is not safe to enter the rich reward area, MCTS-RW and MCTS-Rand both avoid the rich reward area, while MCTS will enter the area as it ignores the presence of the agent (Figure 5F). Comparing these three models across the two environment types allows us to determine the strategy that most accurately approximates participants’ behavior and demonstrate that participants are accounting for the agent’s goal-directed behavior. Through this method, we were able to determine the level of complexity that characterized participants’ planning process.

Results revealed that the model that accounted for the agent’s expected goal-directed behavior (MCTS-RW) fit significantly better than MCTS-Rand in the approach condition $\left(t(79)=7.97,\text{p}=1.98\times 10^{-11},d=1.78\right.$; Figure 5G) and significantly better than MCTS in the avoid condition $(t(79)=7.17,p=7.09\times 10^{-10},d=1.60$; Figure 5G), suggesting that participants were engaging in a multistep planning process that explicitly accounted for the agent’s goal-directed movements and raising the possibility that individual differences in avoidant planning may be explained by the characteristics of this planning process.

### Uncertainty about threatening agents’ decision-making induces avoidant behavior

**Figure 6.**
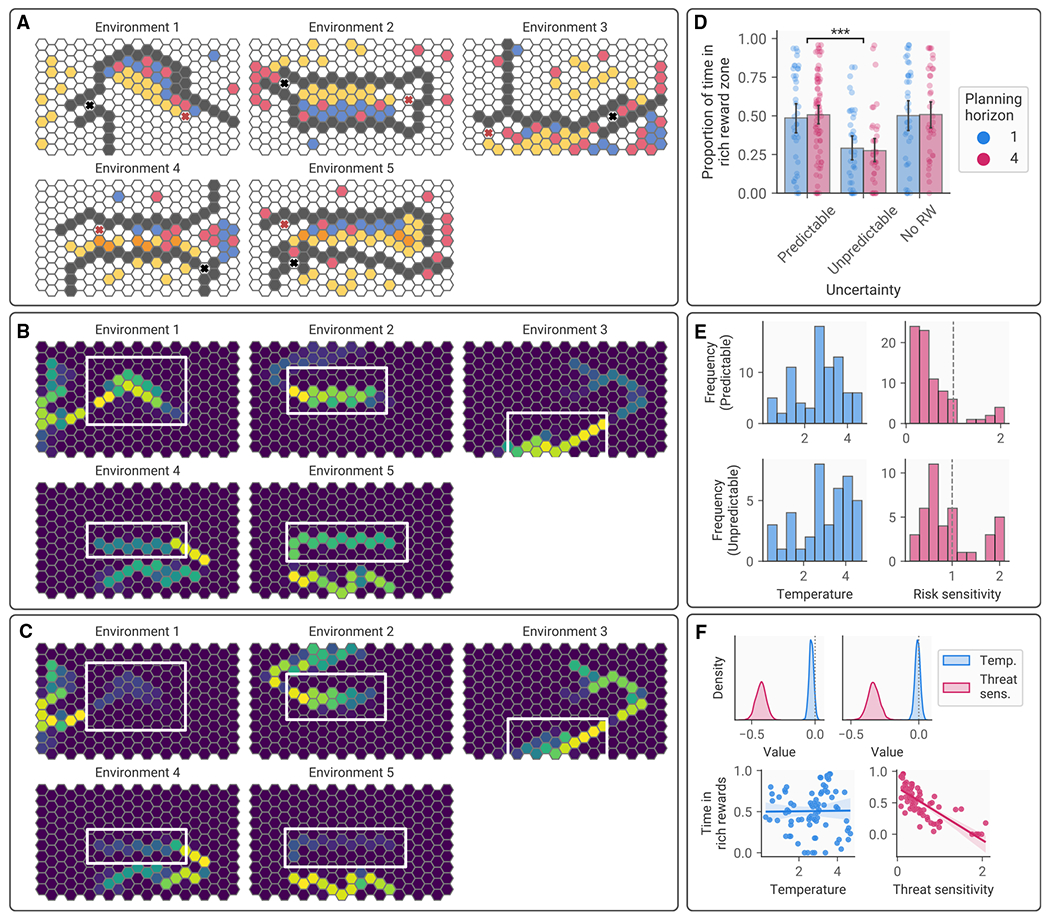
Effects of uncertainty on participants’ behavior (A) Environments used in experiment 3. Yellow, reward; blue, trees; red, red ground; gray, wall. The red and black crosses represent the agent and prey, respectively. (B) State occupancy in the long planning horizon, predictable agent condition. (C) State occupancy in the long planning horizon, unpredictable agent condition, demonstrating greater avoidance of the rich reward zones. (D) Proportion of time spent in rich reward zones (indicated by highlighted rectangular areas) for each condition. Proportions represent the amount of time spent in rich reward zones out of the maximum amount of time that was possible in each environment, with bars representing the mean proportion across participants and error bars representing 95% confidence intervals. ${}^{***}\text{p}<.001$. (E) Distribution of inferred softmax temperature parameter values and threat sensitivity values in the predictable (top) and unpredictable (bottom) conditions for the MCTS-RW planning model. (F) Relationship between model parameters and avoidant. The top shows posterior density estimates for Bayesian regression models evaluating the relationship between model parameters and time spent in the rich reward zone. The bottom shows this relationship in the predictable condition in the form of a scatterplot.

In experiment 2, the agent behaved predictably, selecting actions according to a max policy, and participants were explicitly informed of its reward weights. As a result, it was possible for participants to predict its behavior with high accuracy and adapt their plans accordingly. We reasoned that if this were made more challenging, participants would become more avoidant, for example, becoming less likely to select a patch of rich rewards nearer the agent, even when the agent was unlikely to traverse this region, in favor of a sparsely rewarded patch that was farther from the agent. We also expected that the planning horizon would influence avoidance, with longer planning horizons inducing more uncertainty about encounters (due to a higher number of possible futures to be evaluated) with the agent, leading to more avoidant behavior. To test this, we conducted experiment 3 (n=280; Figure 6A), which manipulated irreducible uncertainty about the predator’s actions (through the predator selecting actions stochastically) and reducible uncertainty about the threatening agent’s preferences (by requiring participants to infer the predator’s preferences) alongside the number of moves made by the participant on each turn (one move or four). This resulted in a 2 (short or long planning horizon) ×3 (predictable, irreducible uncertainty, reducible uncertainty) factorial design. For simplicity, the predator had a consistent preference for the trees only across all conditions, and participants played one practice game initially (in an environment designed to make it impossible for the agent to catch the participant) to demonstrate the agent’s behavioral characteristics.

A between-participants ANOVA revealed a main effect of uncertainty regarding the predator’s actions on the amount of time participants spent in the rich reward zone (F(2,274)=17.70, $p=5.86\times 10^{-8},\eta_{p}^{2}=0.11$; Figure 6D), which planned contrasts indicated was driven by less time being spent in the rich reward zone when the agent behaved unpredictably relative to the condition where it chose actions predictably (t(158)=5.15,p=0.000002,d=0.82; Figures 6B–6D). Contrary to our hypothesis, contrasts indicated there was no significant difference between the predictable agent condition and the condition where no information regarding reward weights was provided (t(162.37)=−0.12,p=.90,d=−0.02; Figure 6D), meaning that participants were less likely to enter the rich reward zone when there was irreducible uncertainty about the agent’s behavior. Further, qualitatively, behavior followed similar patterns across both conditions, indicating that it was not the case that participants followed an equally avoidant but distinct trajectory when this information was not provided. In addition, there was no main effect of planning horizon $(F(1,274)=0.03,\text{p}=.87,\eta_{p}^{2}=0.00$; Figure 6D), indicating that uncertainty induced by the depth of the planning process did not increase avoidance.

### Individual differences in avoidant behavior are not explained by assumptions about threatening agents’ predictability

While our experiments demonstrated a group-level tendency to account for other agents’ behavior optimally, allowing the collection of rewards nearer a threatening agent if the agent’s goal-directed behavior would lead it to avoid the location of these rewards, some participants’ behavior was more avoidant even when the agent behaved predictably. As our results indicated that participants are more avoidant when the agent behaves unpredictably, one potential explanation for these individual differences is variability in participants’ prior expectations of the agent’s predictability. To test this hypothesis, we used a variant of our MCTS planning model incorporating agent action unpredictability, simulating the agent’s actions using a softmax decision rule with an inverse temperature parameter that resulted in different levels of decision noise, alongside a threat sensitivity parameter that modulated the cost of getting caught by the agent.

Results of model fitting in the condition where the agent behaved entirely predictably indicated that softmax temperatures were significantly higher than zero (mean [SD]=2.71[1.08], $t(79)=22.26,\text{p}=8.85\times 10^{-36}$; Figure 6E), indicating some degree of decision noise. We observed a range of threat sensitivity values, with subjects on average underweighting the cost of getting caught (mean [SD]=0.56[0.49]; Figure 6E). To explore the extent to which these two components of the model predicted avoidant behavior, we tested whether the inferred parameter values were correlated with the amount of time spent in the rich reward zone, again focusing on the predictable condition. A Bayesian regression model indicated that threat sensitivity was associated with the degree of avoidance (mean β=−0.43,95%HPDI=[−0.50,−0.35]; Figure 6F), but decision noise was not (mean β=−0.03,95%HPDI=[−0.06,0.01]; Figure 6F). To test this further, we repeated this modeling procedure in the unpredictable condition. Again, threat sensitivity was associated with avoidance (mean β=−0.33,95%HPDI=[−0.42,−0.25]; Figure 6F) while decision noise was not (mean β=−0.01,95%HPDI=[−0.05,0.03]; Figure 6F). To determine the extent to which components of the planning process were influenced by agent unpredictability, we compared parameter values across the predictable and unpredictable conditions. Counter to our expectations, this revealed that inferred decision noise did not differ across conditions (predictable mean [SD]=2.71[1.08], unpredictable mean [SD]=3.02[1.27], t(118)=1.40,p=0.16), but threat sensitivity did (predictable mean [SD]=0.56[0.49], unpredictable mean [SD]=0.93[0.58], t(118)=3.66,p=0.00038), suggesting that people became more sensitive to threat in the unpredictable condition but were not explicitly simulating the predator’s actions in a more unpredictable manner.

## DISCUSSION

The ability to predict other agents’ actions and respond accordingly is vital for ensuring appropriate behavior in a range of social settings. Here, we demonstrate that, in the context of a predator-prey setting, human participants infer virtual threatening agents’ preferences using a model of the social environment and that information about agents’ goal-directed behavior based on these preferences is used when planning to maximize reward gained while avoiding danger.

We found that participants could infer the reward weights of an agent based purely on observations of its behavior. While it is well established that humans are able to infer others’ goals, our findings demonstrate how this occurs at a computational level in a large and complex open environment. Specifically, this was explained by an inverse reinforcement learning model that used Bayesian inference to evaluate hypotheses about the agent’s preferences. Importantly, this model relied on a model of the environment, indicating that participants use an internal model of the world to make inferences about other agents’ preferences. It is also notable that participants did not only learn the agent’s policy, as modern inverse reinforcement learning algorithms typically do,^23–25^ but also learned the weights it placed on different features in the environment, as has been shown in other computational models of social behavior in humans.^8,9^ This enables broad generalization across distinct environments, with different transition structures and feature distributions, as knowing the agent’s reward weights allows its intentions to be inferred across any environment. These findings extend prior work on imitation learning and goal inference^14,16,26^ in humans to more complex, open environments and indicate that these processes not only facilitate social understanding but also allow flexible avoidance of threats. We also note that, while the task was intentionally designed to enable successful learning of reward weights, there was substantial individual variability in accuracy, suggesting that there may be subtle individual differences in the exact strategy used to achieve this.

Our results indicate that human participants are adept at predicting the behavior of freely moving threatening agents. When asked to predict the behavior of another agent in an open virtual environment, all participants were able to do so at levels that were well above chance. While this may appear intuitive, the mechanisms necessary to achieve such accurate prediction are non-trivial. Using computational modeling, we demonstrated that these predictions were best explained by a combination of model-based and model-free strategies, a result reminiscent of previous findings in non-competitive social interactions showing that humans adaptively combine imitation and emulation strategies when learning about others’ behavior,^14^ albeit with the model-based strategy making the strongest contribution. This model-based strategy used information about the agent’s goals to infer its intentions and, thus, its behavior. These results indicate that humans exploit an internal model of their social environment, including knowledge of agents’ goals, to enable flexible avoidance that goes beyond simple stimulus-response strategies. This has important implications, as it indicates that sophisticated model-based planning not only is used to guide first-person decision-making but also can be flexibly deployed to plan from other agents’ perspectives, enabling their likely course of action to be predicted.

When focusing on participants’ own planning, we found that decision-making was best explained by a tree-search planning model that accounted for the agent’s goal-directed behavior, indicating that participants’ knowledge of the agent’s intentions is actively exploited when planning to avoid predation. While it may seem intuitive that people should use this knowledge, our results reveal that such behavior relies upon complex mechanisms involving interactive simulations of multiple agents several steps into the future. This extends prior work demonstrating that tree-search algorithms can approximate human planning behavior^9,10,22,27^ by showing that this process can incorporate simulations of other agents’ goal-directed actions in complex open-world situations and that this multistep interactive planning process enables flexible avoidance. We also found that participants were generally more avoidant when agents behaved less predictably, a finding reminiscent of prior work showing that uncertainty in predator attack locations promotes avoidant behavior,^28^ although this was the case only when unpredictability was induced by random action selection. While we did not observe any effect of the planning horizon, which we expected because a longer planning horizon induces uncertainty by expanding the number of possible futures to be evaluated, this may be because many of these possible futures do not in fact involve getting caught. We also did not find any increase in avoidance when the predator’s reward weights had to be inferred, rather than being provided. However, this may reflect a failure of the manipulation, as participants were generally able to infer these reward weights quickly.

Notably, computational modeling revealed that individual differences in avoidant behavior when the agent behaved predictably were associated with assumptions about the agent’s predictability, with participants who assumed the agent to behave less predictably being more avoidant. Such overestimations of the agent’s unpredictability are not necessarily suboptimal; in situations where only a limited number of moves have been observed it may be rational not to assume the other agent is entirely predictable. Avoidant behavior in this context may also be influenced by participants’ assumptions about their own ability to act optimally or the success of their actions,^29^ although our task was not designed to test this. These results build on a growing literature on the computational mechanisms supporting stimulus-response learning^30–32^ and model-based planning in avoidance^33–35^ by revealing how humans use knowledge of agents’ intentions to facilitate flexible avoidance. In addition, our results extend the extant literature demonstrating how humans make avoidance decisions in response to simple threatening agents.^36–40^ A common theme running through our results is the emphasis on model-based control: across action prediction, goal inference, and avoidance decision-making, participants relied on a model (or simulation) of the environment to avoid predation. The role of model-based control, and internal cognitive maps more generally, in permitting flexible, generalized behavior is becoming increasingly appreciated,^41–44^ and our results demonstrate that the use of an internal model of the world is a hallmark of avoidance when facing dynamic, social threats.

Our findings also build on studies investigating the computational mechanisms supporting social inference, often referred to as mentalizing. Prior work has highlighted the role of model-based planning and simulation in mental state inference^8–10,16,45,46^ and described how humans adaptively engage goal inference strategies in social interactions.^14^ Our results suggest that these mechanisms also enable flexible avoidance of threatening agents in addition to social interactions with other humans. While our results are immediately applicable to environments involving simple threatening agents, we speculate that similar mechanisms may underpin more complex behavior in everyday life. This has clear relevance for our understanding of psychopathology, where conditions such as social anxiety and psychosis are often associated with pathological inferences regarding threat posed by others and their intent to cause harm.^47–49^ In addition, other work has suggested that distortions within cognitive maps of the environment may play a role in pathological anxiety,^29,50,51^ suggesting that our work may also have implications for this condition.

### Limitations of the study

One limitation of our work is that we did not consider complex cognitive hierarchies, as in previous work on simple interactive games, where participants consider a partner’s own social inferences and act in accordance. While the simplicity of our approach made our computational models tractable and is likely to be representative of simple predators that may lack their own complex prospective social planning abilities, future research should consider how deeper interactive planning may support avoidance. It is also notable that the MaxEnt algorithm was unable to infer the agent’s reward weight accurately. It is possible that this is due to the sparsity of the features in our task environment (most states did not contain a salient feature); MaxEnt relies on feature occupancy counts, which may limit its success when there are few features in the environment. Furthermore, the HypTest model was designed to describe the primary computations underpinning reward weight estimation, but by no means does this represent a full account of the mechanisms supporting this behavior. While the model provides a basic mechanistic explanation for reward weight estimation, an interesting challenge for future research will be to determine in more detail how its components function (for example, sampling of candidate reward weights) at both a computational and a neural level and explore how biases in estimation may arise.

It is important to note that, while we took advantage of the predator-prey setting to investigate these processes, our results are not necessarily specific to threat avoidance. It remains an open question whether the mechanisms identified here are specific to avoidance of social agents or represent more domain-general model-based planning apparatus.^52^ In addition, while we have demonstrated the relevance of these mechanisms to avoidance, it is possible that similar mechanisms underpin non-avoidant behavior in the presence of other agents, such as cooperation. Further, the threat used in our experiments was a loss of points (with associated monetary loss). While we have shown previously that loss of points in engaging, game-based tasks can induce subjective anxiety and replicate behavioral patterns observed in response to electric shocks,^31^ it remains a possibility that this was not as aversive as traditional primary aversive stimuli.

We also focused on characterizing the basic foundational mechanisms that provide for effective behavior in a relatively straightforward task setup, but it will be intriguing to explore how these basic mechanisms may adapt to different environments, particularly when the task is made more challenging. In the same vein, our task made it straightforward for participants to build an accurate model of the other agent and its environment. It would also be interesting for future work to explore how the internal model used to guide prediction and planning may become inaccurate and the consequences of this for behavior. Relatedly, our results focus on computational mechanisms at the algorithmic level and do not reveal their neural implementation directly. However, our results do point clearly toward candidate neural mechanisms. It is likely that the computational mechanisms supporting model-based planning in general also subserve avoidance of threatening social agents, given the reliance of our computational models on internal models of the environment. Model-based planning is known to be dependent upon the hippocampus and medial prefrontal cortices,^53–55^ which are thought to represent internal models both of the environment^56,57^ and of abstract relational knowledge,^58^ including social networks.^59^ In addition, our planning models rely on simulations of trajectories through an interactive state space. Given this, it is notable that recent work has highlighted the importance of state reactivation and sequential replay in human model-based control,^60,61^ including in aversive contexts,^35^ which may represent a neural implementation of the prospective simulations upon which our planning models rely.

## STAR★METHODS

### RESOURCE AVAILABILITY

#### Lead contact

Requests for further information regarding the study should be directed to Dr Toby Wise (toby.wise@kcl.ac.uk).

#### Materials availability

This study did not generate new unique reagents.

#### Data and code availability

- All behavioral data from this work has been deposited on the Open Science Framework: https://doi.org/10.17605/OSF.IO/FWGQA (https://osf.io/fwgqa/).
- All original code has been deposited on GitHub: https://doi.org/10.5281/zenodo.8039324 (https://github.com/tobywise/interactive-avoidance).
- Any additional information required to reanalyze the data reported in this paper is available from the lead contact upon request

### EXPERIMENTAL MODEL AND STUDY PARTICIPANT DETAILS

#### Ethical approval

This study was approved by the California Institute of Technology Institutional Review Board.

#### Sample

Participants were recruited through Prolific^62^, and were selected based on being based in the United States and having a 95% approval rate. All participants provided informed consent. For Experiment 1 we recruited 50 participants in each of the three conditions, for Experiment 2 we recruited 80 participants, and for Experiment 3 we recruited 40 participants in each of the six conditions, except for the long horizon, predictable agent condition where we recruited 80 participants. All sample sizes were determined based on effects seen in pilot data. In the event that participants did not complete the full task, or provided data that was incomplete, we continued recruiting until the required number of usable participants was reached. No participants were excluded subsequently. Age and gender of the subjects were not recorded due to a technical issue, but we do not expect this to influence the generalizability of our results.

### METHOD DETAILS

#### Task

Participants completed a task that involved navigating through a 3D virtual environment with the aim of accumulating reward, while avoiding being eaten by a threatening agent. The environment consisted of a 21 X 10 hexagonal grid, in which certain hexagonal cells were removed to create “walls”. Rewards were represented by spinning gold coins, and the environment contained two other features: red ground (cells colored red) and trees (cells with a tree located on them). Participants controlled a robot agent, while the threatening agent was represented by a “blob monster”. The environment was created using Unity and presented online using WebGL. Participants were informed prior to starting that each reward gained would give them 100 points, while being caught by the agent would cause them to lose 1000 points. It was possible to gain a negative number of points, although this would not result in a negative bonus payment. Points were converted into a monetary reward at the end of the task, with each 1000 points being worth £0.2.

The task was split into a number of different “games”, each of which featured a different environment, but participants were informed that the agent was the same across all the environments they encountered. Within each game, the participant and the agent took turns to move around the environment. Participants were first asked to select the cells they wished to move to, before observing their own movements and then seeing the agent make its own moves. If the agent entered the cell occupied by the participant, participants were told that the robot had been eaten and the game ended. Prior to beginning, participants completed a brief tutorial where they were introduced to the different elements of the task and asked to try out each of these elements (for example, making predictions about the agent’s moves). Experiment 3 also included an additional practice environment after the tutorial and before the environments of interest. This was included to demonstrate the agent’s behavior to participants so that they could gauge its level of predictability but was designed to be challenging for participants to infer the agent’s reward weights so that these remained unclear.

In Experiment 1, participants were asked to predict the agent’s movements prior to seeing it move, which was done by asking them to select the cells they expected it to move to on each turn. After making their predictions, participants were asked to rate their confidence in their predictions using a sliding scale ranging from “not at all confident” to “very confident”. An additional financial bonus was provided for correct predictions to incentivize accurate predictions, with 4 predictions being chosen at random at the end of the task and £0.2 awarded for each correct prediction, where the probability of a prediction being chosen was dependent upon reported confidence in the prediction. Participants were also asked to provide estimates of the agent’s preference, which was done using a 9-point scale, where the midpoint was 0 (i.e., no preference), enabling them to rate its likes and dislikes (Figure 1C). In Experiment 2 and Experiment 3 (except the condition where reward weight information was not provided), participants were shown the agent’s preferences prior to beginning the game using gauges that showed how much it liked or disliked each feature in the environment (Figure 1D). In Experiment 1, participants made one move per turn, while the predator made 2, for a total of 10 turns each. In Experiment 2, participants made 4 moves per turn while the predator made 6, with a total of 2 turns each. Finally, in Experiment 3, the number of moves depended on the condition. In the short planning horizon condition, participants made 1 move per turn while the other agent made 2. In the long planning horizon, participants made 4 moves per turn while the predator made 8. In the short horizon condition, each had 12 turns, while in the long condition each had 3 turns. This ensured that the number of total moves was the same across conditions.

In order to determine the agent’s movements, the environment was represented as a Markov Decision Process (MDP), defined by the 4-tuple (𝒮,𝒜,P,R) where 𝒮 represents the set of all possible states, (each of which corresponds to a cell in the grid), 𝒜 represents the actions available to the agent at each state (typically 6 directions of travel, apart from states at the edge of the grid and walls), P represents the probability of transitioning to a given state *s*’ from state s when choosing action *a* (in this case, transitions were fully deterministic), and R is the reward function, indicating the reward available to the agent for taking action a in state s. States were associated with binary features, $f_{1},f_{2},\ldots .\in F$, and we write $f(s)=\left(f_{1}(s),f_{2}(s),\ldots \right)$ as the feature vector for state s. This vector was continually updated to take account of the movement of the prey, and each state could possess any combination of the features, for example taking the value $f(s)=\left[1 0 1\right]$ if the cell represented by state s was occupied by both trees and the prey. The agent was considered to have a vector r of reward weights such that the net reward associated with state s derived from the dot product $f(s)\cdot r=\sum_{i}f_{i}(s)r_{i}$ between the features and weights. When moving around the environment, the agent’s reward was determined by $R(s,a)=f\left(s^{\prime }\right)\cdot r$ for the state $s^{’}$ (deterministically) entered when taking action *a* in state *s*,. In practice, reward weights were set to either 0 or 1. The predator was forced to move on each state, and therefore if it reached a preferred state, it would subsequently move to other preferred states rather than staying in one place.

The movements of the agent were determined by solving for the optimal value function within this MDP using value iteration, i.e., iteratively applying the update equation (assuming that the prey would stay still):

(Equation 1) $$V_{k+1}(s)=\underset{a}{max}\;\left(R(s,a)+\gamma \sum_{s^{\prime }}P\left(s^{\prime }|s,a\right)V_{k}\left(s^{\prime }\right)\right)$$

For each state *s* in the MDP (where a represents a given action, R(s,a) represents the reward gained by taking action *a* in state s,γ represents a discount factor, and k represents the current iteration. The Q value of each action depends upon the immediate reward received following its selection in addition to the current value estimate of the next state reached:

(Equation 2) $$Q(s,a)=R(s,a)+\gamma \sum_{s^{\prime }}P\left(s^{\prime }|s,a\right)V_{k}\left(s^{\prime }\right)$$

The discount factor γ was set to 0.9 to provide a balance between optimality and computation time and the algorithm was terminated after 500 iterations. The agent’s action selection differed across the experimental conditions. In the majority of conditions (all except the unpredictable condition in Experiment 3), actions were selected using a max strategy (i.e. selecting the action in the current state with the maximum Q value). In other conditions, a softmax decision rule was used instead to engender unpredictability in the agent’s behavior.


(Equation 3) $$P_{t}(s,a)=\frac{e^{\left(Q_{t}(s,a)/\tau \right)}}{\overset{N}{\underset{i=1}{\Sigma }}e^{\left(Q_{t}\left(s,a_{i}\right)/\tau \right)}}$$

Where N is the number of actions available in the current state and τ is a temperature parameter, which was fixed at 1 to make the agent behave in a way that was unpredictable, but not entirely random.

### QUANTIFICATION AND STATISTICAL ANALYSIS

#### Regression models

To assess the development of prediction accuracy and confidence over the course of the task, we used Bayesian regression models implemented in PyMC3^63^. These models characterized the dependent variable (either probability of being correct or confidence ratings) as a linear combination of an intercept, the game number and the trial number. Models used a hierarchical non-centered specification, where the participant-level parameter for predictor k was determined by:

(Equation 4) $$\beta_{\text{subject}}(k)=\mu_{\text{group}}(k)+\sigma_{\text{group}}(k)\cdot \varepsilon_{\text{subject}}(k)$$

where ε is a participant-level offset parameter. For the model predicting prediction accuracy, the model used a Bernoulli likelihood for the observations, while a Beta likelihood was used for the model predicting confidence.

#### Action prediction models

To explain the computational mechanisms supporting participants’ ability to predict the agent’s upcoming movements, we fit a series of decision-making models. The first model family was a simple policy learning model that learned the action that the agent tended to take (i.e., which of the 6 actions, with no regard for the state it currently occupied). This model updated its expectation about the agent’s likely next move according to a prediction error:

(Equation 5) $${\hat{\pi }}_{t}(a)={\hat{\pi }}_{t-1}(a)+\alpha_{t}\cdot \left(\delta_{a,a_{t;obs}}-{\hat{\pi }}_{t-1}(a)\right)$$

where ${\hat{\pi }}_{t}(a)$ is the estimate of the probability of performing action a after observing the action on trial $t,a_{t;\text{obs}}$ is the action actually observed on that trial and $\alpha_{t}$ is a learning rate parameter that scaled the effect of each prediction error. This was designed to decrease with increasing numbers of observations, and was adjusted on each trial according to:

(Equation 6) $$\alpha_{t}=\alpha_{t}\cdot n_{t}^{-\lambda }$$

With n representing the number of observations and λ being a decay parameter that was estimated alongside the starting value of α.

We also extended this model to account for correlations among action values, as adjacent action values are likely to be more similar than non-adjacent ones due to them typically leading to similar future states. To achieve this, we convolved the observed action choice (a one-hot vector representing the one chosen action on the current trial) with a squared exponential kernel.


(Equation 7) $$k\left(x,x_{⊤}\right)=exp\;\left[-\frac{{\left(x-x_{T}\right)}^{2}}{2{ℒ}^{2}}\right]$$

This resulted in the “outcome” of the trial (i.e., the chosen action) being generalized to adjacent actions, as if they had themselves been partially chosen through convolution with this kernel.


(Equation 8) $${\hat{a}}_{t;obs}=k\left(a_{t;obs},a_{t;obs}\right)$$

Where $a_{t;obs}$ is a one-hot vector representing the action chosen by the agent on trial t. The estimate of $\hat{\pi }$ is then updated according to Equation 5, using ${\hat{a}}_{obs}(a)$ in place of $\delta_{a,a_{t;obs}}$. The length scale parameter ℒ was fixed at 0.02 for model fitting. Values were initialized at zero, and learned values persisted across different games.

The final variant of the policy learning model was one that simply assumed the agent would repeat its previous action, which was achieved by setting the learning rate α to 1 and removing the learning rate decay.

We also fit a model that predicted the agent’s moves based on its inferred goals, using an explicit model of the task. This was achieved by determining the optimal policy using value iteration (Equation 1) and making predictions assuming the agent would select a sequence of actions using a max policy. Finally, we fit models that combined the policy learning model variants with the goal inference model. These models used the $\hat{\pi }$ values estimated by each model, scaled to the range 0−1 to ease interpretation of resulting weights on the values from each model:

(Equation 9) $$\hat{\pi }=\frac{\hat{\pi }-min(\hat{\pi })}{max(\hat{\pi })-min(\hat{\pi })}$$

These were then weighted according to weighting parameter W (which could take values from 0 to 1).


(Equation 10) $${\hat{\pi }}_{\text{combined}}(a)=W\cdot {\hat{\pi }}_{\text{goal}}(s,a)+(1-W)\cdot {\hat{\pi }}_{\text{policy}}(a)$$

This provided a prediction about the agent’s preferred action that combined information from policy learning and goal inference models. There were 3 variants of the combined model, combining the goal inference model with each of the three policy learning models. For all models, Q values were transformed into choice probabilities using a softmax function with a temperature parameter value of 1 (Equation 3), to account for uncertainty in participants’ predictions. Model fit was determined according to the log likelihood of the model with a categorical likelihood function.


(Equation 11) $$-L(\theta )=-\sum_{t=1}^{N}log\left({\hat{\pi }}_{\text{combined}}\left(a_{t;\text{obs}}\right)\right)$$

Where ${\hat{\pi }}_{\text{combined}}\left(a_{t;\text{obs}}\right)$ is the probability of the chosen action on trial t up to total predictions N. Parameters θ were the learning rate α in the policy learning models and the weighting parameter W in the combined models, and were estimated using differential evolution in SciPy. In addition, we calculated the accuracy of categorical predictions made by each model, and the Bayesian Information Criterion (BIC) of the model as an index of model fit that accounted for model complexity.

### Inverse reinforcement learning models

Participants’ ratings of the agent’s reward weights were modelled using a series of inverse reinforcement learning models. While the goal of standard reinforcement learning is to find a policy that maximizes long-run reward given an MDP with a known reward function, inverse reinforcement algorithms seek to infer the reward function of an MDP (or for some algorithms an agent’s policy, or its reward weights) given observations of an agent’s actions within that MDP. We note that the models described here are not designed to be biologically plausible but are instead intended to illustrate the fundamental computational principles underpinning goal inference. The reward weights represent the agent’s preferences for the features in the environment and can be positive or negative, representing a like or dislike of the feature respectively, and the goal of these models was to estimate these values.

The simplest of these was a model-free strategy based on feature occupancy counts, based on the assumption that the agent would spend more time in state containing the features it preferred. The estimated reward weights r for each of the features f∈F were therefore calculated by:

(Equation 12) $$r_{i}=\sum_{t}f_{i}\left(s_{t}\right)$$

Where $s_{t}$ each state occupied by the agent and $f_{i}\left(s_{t}\right)$ is a binary indicator of the $i^{\text{th}}$ feature’s presence in state $s_{t}$. The resulting individual feature weights were used to compose the vector of feature weights r. For all model-free algorithms, the reward weight estimation process was repeated at each step, with the feature map updated to account for the prey’s movements, and the resulting feature weights were then summed across all time steps before being normalized, as described below.

The next model was also a model-free method that estimated the agent’s reward weights based on its direction of travel. This summed the features that the agent would encounter in the states $s^{\prime }$ it would occupy if it were to continue in its current direction of travel (i.e., repeating its prior action until it reached the edge of the grid, at which point the accumulation of feature counts ceased), repeating this process at each state s it was observed in. This represents a simplistic model-free method for estimating the predator’s preferences based on its direction. The weight of each feature at each time step t was calculated as follows:

(Equation 13) $$r_{i}=\sum_{t}\sum_{s^{\prime }\in dir\left(s_{t},a_{t;obs}\right)}f_{i}\left(s^{\prime }\right)$$

Where $dir\left(s_{t},a_{t;obs}\right)$ is the set of states that would be traversed starting from state $s_{t}$ and carrying on in direction $a_{t;obs}$ until the end of the grid.

We also extended this to account for the relative frequency of features encountered along the agent’s direction of travel compared to alternative directions. This involved repeating the process of feature counting for all alternative directions of travel (i.e., repeating the other 5 actions available in the current state, and continuing to the edge of the grid) and summing the result.

Feature weight vectors for both the observed and alternative paths were then normalized as follows:

(Equation 14) $$r_{\text{norm}}=\frac{r}{\underset{i=1}{\Sigma }r_{i}}$$

Relative feature weights for the observed versus alternative trajectories were then calculated:

(Equation 15) $$r=r_{\text{norm}}^{\text{obs}}-r_{\text{norm}}^{\text{alt}}$$

Where $r_{\text{norm}}^{\text{obs}}$ are is the normalized feature counts from the observed direction of travel and $r_{\text{norm}}^{\text{alt}}$ is the equivalent for the alternative directions of travel. One limitation of all these model-free methods is that they have difficulty accounting for the prey feature, as they rely on accumulated feature counts within a trajectory; as the prey can only be encountered once, these approaches will tend to underweight the prey. In addition, being model-free they have no ability to represent the prey’s future moves, and therefore assume the prey will remain in its current position.

We compared these model-free methods against two model-based inverse reinforcement learning algorithms. The first was a variant of Infinite Time Horizon Maximum Causal Entropy^64^ (MaxEnt). This is an extension of the Maximum Causal Entropy^20^ and Maximum Entropy^21^ inverse reinforcement learning algorithms but applied to MDPs with no clear terminal states, as the environments used in these experiments could be navigated freely for the number of moves allowed, with no absorbing states present. This family of algorithms leverages the principle of maximum entropy to resolve uncertainty regarding the true reward weights, given IRL problems are typically ill-posed with multiple potential solutions. Accordingly, MaxEnt prefers a policy that matches observed behavior but is most uncertain otherwise. Exhaustive details on these algorithms are provided in the respective original papers, and here we provide an overview of the basic algorithm used here.

This algorithm seeks to infer an agent’s reward weights based on observations of its actions within a given fully observable MDP based on the principle of feature matching; this constrains the proposed reward weights based on the condition that they result in a policy that encounters features with the same frequency as the observed behavior of the agent. The algorithm thus comprises two steps: 1) Given an estimate of the agent’s reward weights $\hat{r}$, identify a policy $\hat{\pi }$ for the MDP using standard reinforcement learning; 2) update the estimated reward weights $\hat{r}$ according to how accurately behavior under policy $\hat{\pi }$ matches the features of the observed behavior. We elected to use an infinite horizon variant of MaxEnt, as although the predator was given a fixed number of steps there were no clear terminal states. While there may be some minor time-dependence in the predator’s policy, environments were designed to minimize this, and the fact that the predator did not consume features on encountering them also served to limit time-dependence.

More specifically, the algorithm starts with a randomly chosen estimate of $\hat{r}$ (which we set to zero for each feature) and solves the MDP based on the reward function determined by these reward weights using soft value iteration^64^ to provide the policy $\hat{\pi }$. This adapts the Q value update equation of standard value iteration (Equation 1) to use soft value estimates:

(Equation 16) $$Q(s,a)=R(s,a)+\gamma \sum_{s^{\prime }}P\left(s^{\prime }|s,a\right)V_{k}^{\text{soft}}\left(s^{\prime }\right)$$

Where $V_{k}^{\text{soft}}$ for a given state s is calculated by applying a form of softmax function to the vector of Q estimates representing the value of valid actions from that state:

(Equation 17) $$V_{k+1}^{\text{soft}}(s)={softmax}_{VI}\left(\{Q(s,a){\}}_{a}\right)$$

Where the ${\mathrm{softmax}}_{\mathrm{VI}}$ function is defined as follows^20^ for a vector of values **x**:

(Equation 18) $${softmax}_{VI}(x)=log\sum_{x}e^{x}$$

The policy $\hat{\pi }$ was determined by using a standard softmax function (Equation 3, with temperature set to 1) to calculate choice probabilities, this is used to derive expected state visitation counts under this policy. Visitation counts D for each state s are initialized at 0, and the following update is run iteratively for each state s, each action a available from s and each subsequent state $s^{\prime }$ that can be reached by taking action a in state s (as the MDPs used here were deterministic, only one state could be reached through each state action pair).


(Equation 19) $$D_{k+1}\left(s^{\prime }\right)=D_{k}\left(s^{\prime }\right)+D_{k}(s)\cdot \hat{\pi }(a,s)\cdot P\left(s^{\prime }|a,s\right)$$

Where k represents the current iteration. This is run until convergence, where estimated visitation counts change minimally between iterations. These state visitation counts can then be used to calculate feature expectations ${ℱ}_{\overset{ˆ}{R}}$ according to reward weights $\hat{R}$ and associated policy $\hat{\pi }$:

(Equation 20) $${ℱ}_{\overset{ˆ}{r}}=\sum_{s}^{𝒮}D(s)\cdot f(s)$$

Observed feature counts ${ℱ}_{𝒪}$ are then calculated as the normalized frequency of each feature in the set of observed states visited by the agent ${𝒮}_{𝒪}$:

(Equation 21) $${ℱ}_{𝒪}=\frac{1}{\left|{𝒮}_{𝒪}\right|}\sum_{s_{𝒪}}^{{𝒮}_{𝒪}}f\left(s_{𝒪}\right)$$

The vector difference between observed feature counts ${ℱ}_{𝒪}$ and expected feature counts ${ℱ}_{\overset{ˆ}{r}}$ for all features can then be used as an approximation of the accuracy of the current reward weight estimate.


(Equation 22) $$\delta_{ℱ}={ℱ}_{𝒪}-{ℱ}_{\overset{ˆ}{R}}$$

This error can then be used to estimate the true reward weights r, where the estimate $\hat{r}$ is updated on each iteration k of the optimization process through:

(Equation 23) $${\hat{r}}_{k+1}={\hat{r}}_{k}+\alpha_{k}\cdot \delta_{ℱ}$$

Where $\alpha_{k}$ is a learning rate parameter that updates on each trial according to Equation 6. This is repeated until convergence, or when a pre-specified maximum number of iterations (set to 1000 here) is reached. Because MaxEnt relies on comparing state visitation counts within an entire multi-step trajectory following a single policy, it is unable to account for changing features at each time step. In principle, it would be possible to use single-step trajectories which would allow for movement of the prey at each step, however this would not provide sufficient information for the algorithm to determine feature weightings as feature counts would be based on only a few steps at most. Therefore, in the reward weight condition where the threatening agent had a preference for the robot, it is unable to infer valid reward weights.

Finally, we defined a model-based algorithm that inferred the agent’s reward weights through a process we refer to as hypothesis testing (HypTest), related to prior work on hypothesis testing in human decision-making^15^. This approach uses Bayesian inference to determine the likelihood of a given set of reward weights.


(Equation 24) P(weights∣behavior)=P(behavior∣weights)P(weights)

In order to estimate the likelihood of behavior given a set of weights (P(behavior∣weights)), we determine the optimal policy for the MDP according to these weights and use this to determine the likelihood of each action in a given state. While this can be achieved using any valid method for computing action probabilities (for example, dynamic programming or tree search) we use the successor representation (SR)^17^ to estimate the optimal policy. This is due to its computational simplicity and ability to adapt in response to changes in reward functions, and we do not suggest that human participants are necessarily using the SR. The SR is computed using a matrix of state expectancies (i.e., the discounted likelihood of visiting each state in the future based on the state currently occupied, based on the objective transition function for the MDP according to a uniform policy), and a vector of rewards available in each state. Note that we assume full knowledge of the objective transition function and use this to derive expectancies, instead of using a learned state expectancy matrix as is commonly done when using the SR^18,65^. In order to estimate action values, we represent the state occupancy matrix in terms of state action pairs instead of states alone^18^$\left(M\left(\{s,a\},\left\{s^{\prime },a^{\prime }\right\}\right)\right)$ and rewards based on rewards associated with each state-action pair (R(s,a)). Thus, the Q value for each action in the current state could be calculated by taking the inner product of the row in M corresponding to that state action pair (M({s,a},{*})) and the reward rector *R*:

(Equation 25) Q(s,a)=M({s,a},{*})×R

Q values were estimated separately for each step in the agent’s trajectory by repeatedly applying Equation 25 using an updated reward vector R, accounting for the prey’s movements, such that the resulting reward weights are estimated over the entire set of observations. To convert Q values to action probabilities, we used a softmax function (Equation 3) with the temperature parameter set to 0.083 (the mean inferred softmax value in Experiment 3). Following prior work^15^, we used Markov Chain Monte Carlo (MCMC) sampling to approximate the posterior distribution over reward weights using the action probabilities described above combined with a flat generalized beta prior over each reward weight (rescaled to the range [−1, 1]). We used No U-Turn Sampling^66^, a form of Metropolis-Hastings algorithm, implemented in NumPyro^67^ with 2000 samples. We used this approach as an implementation of sampling more generally, and do not suggest that this is the exact algorithm being used by human participants. More generally, although sampling is one plausible method by which human participants may estimate reward weights, alternative parameter estimation methods may be equally effective in explaining human behavior. For the purposes of model comparison, we used the mean of the posterior distributions as a point estimate of the other agent’s preferences. Importantly, because the HypTest algorithm determines the most likely action at each time step independently of all others (in contrast to MaxEnt, which uses an entire trajectory according to a single policy), it is able to estimate valid reward weights even in the presence of changing features, such as the robot feature.

Predictions from the models were scaled to the same range as the subjects’ predictions −4 to +4 to aid comparison between models. To determine the best fitting model, we simulated predictions from each model across a range of hyperparameter values and calculated the adjusted $R^{2}$ of each model to provide a measure of model fit accounting for complexity.


(Equation 26) $$\text{adjusted}R^{2}=1-\left(1-R^{2}\right)\frac{n-1}{n-p-1}$$

Where n is the number of observations (collapsing across trials and participants) and p is the number of parameters in the model. We also calculated the Bayesian Information Criterion (BIC) as an additional index of model fit.

#### Interactive planning models

Participants’ own movements in the task were modelled using a series of planning models. For this purpose, the task was represented as a 1^st^-order interactive MDP (i.e., the modelled participant, as prey, accounts for the actions of the agent, as predator, but does not account for the predator’s expectations of the prey’s actions) where each state is defined jointly by the positions of the agent and the prey. This results in a large state space 210^2^ states) which cannot be solved easily by dynamic programming approaches. Instead, we used Monte Carlo Tree Search (MCTS) as to approximate the optimal policy online, specifically the Upper Confidence Bound for Trees (UCT) variant^68^ (for simplicity, we refer to our approach as MCTS). The MCTS family of algorithms approximate the optimal policy in a given state using sampling of potential trajectories and gained rewards. The algorithm does not require an explicit model of the world like dynamic programming solutions but does require the ability to simulate the outcome of actions. This algorithm is described extensively elsewhere^68–70^, and here we focus on the specific extensions made to enable interactive planning (i.e. planning that accounts for another agent).

In addition to non-interactive MCTS that ignores the presence of the agent, simulating only the prey’s actions and reward gained, we also extend the algorithm to simulate actions of the agent. This approach is often taken when seeking to optimize behavior in multi-player games^69,70^, and in these scenarios the opponent’s policy is also optimized as part of the algorithm (i.e. the opponent’s simulated actions are selected using the same UCT rule as the player’s). Here, we instead simulate the agent’s actions using two different approaches to enable us to test hypotheses about the computational mechanisms supporting interactive avoidance planning. In the first model variant (MCTS-Rand), we determined the agent’s chosen action in the simulation process randomly, assuming the agent is known to exist, but the prey has no knowledge about its policy. The final model (MCTS-RW) simulates the behavior of the agent according to its policy which is estimated based on its known reward weights. The model assumes that the agent chooses the action with the highest Q value, but we also extend the model to simulate the agent’s actions according to a softmax rule with a variable temperature parameter τ (Equation 3). This equates to fully interactive planning, where the simulation process accounts for both the states visited by the prey and the actions of the agent. The agent’s policy was determined using value iteration (Equation 1) using the objective reward weights provided to the participant, and the simulation was run for as many steps as remained at the current trial. We note here that this process only accounts for the simplest of social inferences, referred to variously as Level 0 or Level 1 theory of mind, where the prey is accounting for the agent’s actions in its planning, but not accounting for the agent planning based on its own expectations of the prey’s actions.

As MCTS is a stochastic, simulation-based approach, the likelihood function for these models is not possible to calculate analytically. Therefore, in order to determine how well these models fit the data, we used Inverse Binomial Sampling (IBS)^22^ as a robust method for estimating the likelihood based on repeated runs of the model. We repeated this process 16 times per model to reduce the variance of the estimate. For analyses estimating the softmax temperature parameter across the sample, we ran the model fitting procedure across a grid of 10 candidate parameter values between 0 and 1, determining the best fitting value according to its loglikelihood.

To estimate parameters for the winning planning model, we used simulation-based inference (SBI)^71,72^. Specifically, we used neural posterior estimation (NPE) as implemented in the SBI toolbox (https://www.mackelab.org/sbi/), an approach that is able to estimate parameters of stochastic models in a computationally efficient manner. The SBI procedure involves producing simulated datasets using the chosen model across a range of parameter values (in our case 20,000 such datasets using parameter values drawn uniformly at random). Subsequently, a neural network is trained on this data to learn a nonlinear function mapping observed behavior to the parameters that generated it. By then applying this network to subjects’ observed behavior, we were able to estimate parameters of the model for individual subjects.

We note that this constitutes a deviation from our preregistered analysis plan, which originally indicated that we would estimate softmax temperature values using a grid search procedure. However, advances in model-fitting since writing the preregistration allowed us to additionally estimate a threat sensitivity parameter to determine whether variation in behavior was best explained by individual differences in inferred unpredictability of the predator or threat sensitivity. The additional complexity of this analysis would make a grid search estimation approach less computationally feasible, but it is feasible with SBI.

### Model and parameter recovery

For each of our modelling analyses, we conducted model and parameter recovery analyses to determine how accurately we were able to distinguish between candidate models and to estimate the values of parameters within these models. All results from these analyses are included in Supplementary Material.

For the action prediction models, we generated 40 simulated datasets per model with parameters drawn from uniform distributions. For each model, we calculated the proportion that were best fit by each of the candidate models. We also calculated Pearson correlations between the parameter values used to simulate the data and those estimated based on the resulting simulated datasets.

For the inverse reinforcement learning models, we generated 150 simulated datasets from each model and estimated model fit for each candidate model across these datasets. We also estimated the stability of these results by repeating the procedure on randomly-selected subsets of 50 simulated datasets and then calculating the proportion of datasets in which each model was chosen as the best fitting model.

For the planning models, we generated 80 simulated datasets for each model. We then fit each candidate model to this simulated data, calculating the proportion of simulated datasets best fit by each model.

### ADDITIONAL RESOURCES

The methodology and hypotheses for the experiments reported here were preregistered on the Open Science Framework (https://osf.io/bgr4v).

## Supplementary Material

1

## Figures and Tables

**Table:** KEY RESOURCES TABLE

REAGENT or RESOURCE	SOURCE	IDENTIFIER
Deposited data		
Human behavioral data	Open Science Framework	https://doi.org/10.17605/OSF.IO/FWGQA
Experimental models: Organisms/strains		
Human participants	Prolific	https://www.prolific.co/
Software and algorithms		
Custom analysis code	GitHub	https://doi.org/10.5281/zenodo.8039324
Unity	Unity Technologies	https://unity.com/
Firebase	Google	https://firebase.google.com/

## Highlights

- Human participants infer other agents’ goals using a model of the interactive environment
- Predictions about other agents’ behavior are achieved by using knowledge of their goals
- Participants’ behavior is explained by a model that accounts for other agents’ behavior

## References

[1] FeldmanHall O, and Shenhav A (2019). Resolving uncertainty in a social world. Nat. Human Behav 3, 426–435. 10.1038/s41562019-0590-x. 31011164 PMC6594393

[2] Barrett HC (2015). Adaptations to predators and prey. The handbook of evolutionary psychology, 200–223.

[3] Bargh JA, Schwader KL, Hailey SE, Dyer RL, and Boothby EJ (2012). Automaticity in social-cognitive processes. Trends Cognit. Sci 16, 593–605. 10.1016/j.tics.2012.10.002. 23127330

[4] Courbin N, Loveridge AJ, Macdonald DW, Fritz H, Valeix M, Makuwe ET, and Chamaillé-Jammes S (2016). Reactive responses of zebras to lion encounters shape their predator–prey space game at large scale. Oikos 125, 829–838. 10.1111/oik.02555.

[5] Blakemore S-J, and Decety J (2001). From the perception of action to the understanding of intention. Nat. Rev. Neurosci 2, 561–567. 10.1038/35086023. 11483999

[6] Arora S, and Doshi P (2021). A survey of inverse reinforcement learning: Challenges, methods and progress. Artif. Intell 297, 103500. 10.1016/j.artint.2021.103500.

[7] Wu CM, Vélez N, and Cushman F (2021). Representational Exchange in Human Social Learning: Balancing Efficiency and Flexibility. 10.31234/osf.io/rm52c.

[8] Baker CL, Jara-Ettinger J, Saxe R, and Tenenbaum JB (2017). Rational quantitative attribution of beliefs, desires and percepts in human mentalizing. Nat. Human Behav 1, 0064–110. 10.1038/s41562-017-0064.

[9] Hula A, Vilares I, Lohrenz T, Dayan P, and Montague PR (2018). A model of risk and mental state shifts during social interaction. PLoS Comput. Biol 14, e1005935. 10.1371/journal.pcbi.1005935. 29447153 PMC5831643

[10] Hula A, Montague PR, and Dayan P (2015). Monte Carlo Planning Method Estimates Planning Horizons during Interactive Social Exchange. PLoS Comput. Biol 11, e1004254. 10.1371/journal.pcbi.1004254. 26053429 PMC4460182

[11] Na S, Chung D, Hula A, Perl O, Jung J, Heflin M, Blackmore S, Fiore VG, Dayan P, and Gu X (2021). Humans use forward thinking to exploit social controllability. Elife 10, e64983. 10.7554/eLife.64983. 34711304 PMC8555988

[12] Hampton AN, Bossaerts P, and O’Doherty JP (2008). Neural correlates of mentalizing-related computations during strategic interactions in humans. Proc. Natl. Acad. Sci. USA 105, 6741–6746. 10.1073/pnas.0711099105. 18427116 PMC2373314

[13] Yoshida W, Dolan RJ, and Friston KJ (2008). Game Theory of Mind. PLoS Comput. Biol 4, e1000254. 10.1371/journal.pcbi.1000254. 19112488 PMC2596313

[14] Charpentier CJ, Iigaya K, and O’Doherty JP (2020). A Neuro-computational Account of Arbitration between Choice Imitation and Goal Emulation during Human Observational Learning. Neuron 106, 687–699.e7. 10.1016/j.neuron.2020.02.028. 32187528 PMC7244377

[15] Dasgupta I, Schulz E, and Gershman SJ (2017). Where do hypotheses come from? Cognit. Psychol 96, 1–25. 10.1016/j.cogpsych.2017.05.001. 28586634

[16] Baker CL, Saxe R, and Tenenbaum JB (2009). Action understanding as inverse planning. Cognition 113, 329–349. 10.1016/j.cognition.2009.07.005. 19729154

[17] Dayan P (1993). Improving Generalization for Temporal Difference Learning: The Successor Representation. Neural Comput. 5, 613–624. 10.1162/neco.1993.5.4.613.

[18] Momennejad I, Russek EM, Cheong JH, Botvinick MM, Daw ND, and Gershman SJ (2017). The successor representation in human reinforcement learning. Nat. Human Behav 1, 680–692. 10.1038/s41562-017-0180-8. 31024137 PMC6941356

[19] Bloem M, and Bambos N (2014). Infinite time horizon maximum causa entropy inverse reinforcement learning. In 53rd IEEE Conference on Decision and Control, pp. 4911–4916. 10.1109/CDC.20147040156.

[20] Ziebart BD, Bagnell JA, and Dey AK (2010). Modeling Interaction via the Principle of Maximum Causal Entropy.

[21] Ziebart BD, Maas A, Bagnell JA, and Dey AK (2008). Maximum Entropy Inverse Reinforcement Learning. In Proceedings of the 23rd Nationa Conference on Artificial Intelligence - Volume 3 AAAI’08 (AAAI Press)), pp. 1433–1438.

[22] van Opheusden B, Acerbi L, and Ma WJ (2020). Unbiased and efficient log-likelihood estimation with inverse binomial sampling. PLoS Comput. Biol 16, e1008483. 10.1371/journal.pcbi.1008483. 33362195 PMC7758077

[23] Ho J, and Ermon S (2016). Generative Adversarial Imitation Learning. In Advances in Neural Information Processing Systems (Curran Associates, Inc.).

[24] Qureshi AH, Boots B, and Yip MC (2019). Adversarial imitation via variational inverse reinforcement learning. Preprint at arXiv, 1809.06404. 10.48550/arXiv.1809.06404.

[25] Fu J, Luo K, and Levine S (2018). Learning robust rewards with adversarial inverse reinforcement learning. Preprint at arXiv, 1710.11248. 10.48550/arXiv.1710.11248.

[26] Collette S, Pauli WM, Bossaerts P, and O’Doherty J (2017). Neural computations underlying inverse reinforcement learning in the human brain. Elife 6, e29718. 10.7554/eLife.29718. 29083301 PMC5662289

[27] Huys QJM, Eshel N, O’Nions E, Sheridan L, Dayan P, and Roiser JP (2012). Bonsai Trees in Your Head: How the Pavlovian System Sculpts Goal-Directed Choices by Pruning Decision Trees. PLoS Comput. Biol 8, e1002410. 10.1371/journal.pcbi.1002410. 22412360 PMC3297555

[28] Qi S, Cross L, Wise T, Sui X, O’Doherty J, and Mobbs D (2020). The Role of the Medial Prefrontal Cortex in Spatial Margin of Safety Calculations. Preprint at bioRxiv 10.1101/2020.06.05.137075. PMC11340276 38997158

[29] Zorowitz S, Momennejad I, and Daw ND (2020). Anxiety, Avoidance, and Sequential Evaluation. Comput. Psychiatr. Psychol 4, 1–17. 10.1162/CPSY_a_00026. PMC8143038 34036174

[30] Wise T, Michely J, Dayan P, and Dolan RJ (2019). A computational account of threat-related attentional bias. PLoS Comput. Biol 15, e1007341. 10.1371/journal.pcbi.1007341. 31600187 PMC6786521

[31] Wise T, and Dolan RJ (2020). Associations between aversive learning processes and transdiagnostic psychiatric symptoms in a general population sample. Nat. Commun 11, 4179. 10.1038/s41467020-17977-w. 32826918 PMC7443146

[32] Tzovara A, Korn CW, and Bach DR (2018). Human Pavlovian fear conditioning conforms to probabilistic learning. PLoS Comput. Biol 14, e1006243. 10.1371/journal.pcbi.1006243. 30169519 PMC6118355

[33] Lockwood PL, Klein-Flügge MC, Abdurahman A, and Crockett MJ (2020). Model-free decision making is prioritized when learning to avoid harming others. Proc. Natl. Acad. Sci. USA 117, 27719–27730. 10.1073/pnas.2010890117. 33055212 PMC7959560

[34] Wang O, Lee SW, O’Doherty J, Seymour B, and Yoshida W (2018) Model-based and model-free pain avoidance learning. Brain Neurosci. Adv 2, 2398212818772964. 10.1177/2398212818772964. 30370339 PMC6187988

[35] Wise T, Liu Y, Chowdhury F, and Dolan RJ (2021). Model-based aversive learning in humans is supported by preferential task state reactivation. Sci. Adv 7, eabf9616. 10.1126/sciadv.abf9616. 34321205 PMC8318377

[36] Qi S, Hassabis D, Sun J, Guo F, Daw N, and Mobbs D (2018). How cognitive and reactive fear circuits optimize escape decisions in humans. Proc. Natl. Acad. Sci. USA 115, 3186–3191. 10.1073/pnas.1712314115. 29507207 PMC5866541

[37] Silston B, Wise T, Qi S, Sui X, Dayan P, and Mobbs D (2020). Neural encoding of socially adjusted value during competitive and hazardous foraging. Preprint at bioRxiv. 10.1101/2020.09.11.294058. PMC8446065 34531399

[38] Mobbs D, Petrovic P, Marchant JL, Hassabis D, Weiskopf N, Seymour B, Dolan RJ, and Frith CD (2007). When Fear Is Near: Threat Imminence Elicits Prefrontal-Periaqueductal Gray Shifts in Humans. Science 317, 1079–1083. 10.1126/science.1144298. 17717184 PMC2648508

[39] Fung BJ, Qi S, Hassabis D, Daw N, and Mobbs D (2019). Slow escape decisions are swayed by trait anxiety. Nat. Human Behav 3, 702–708. 10.1038/s41562-019-0595-5. 31110337 PMC7755102

[40] Bach DR, Guitart-Masip M, Packard PA, Miró J, Falip M, Fuentemilla L, and Dolan RJ (2014). Human Hippocampus Arbitrates Approach-Avoidance Conflict. Curr. Biol 24, 541–547. 10.1016/j.cub.2014.01.046. 24560572 PMC3969259

[41] Behrens TEJ, Muller TH, Whittington JCR, Mark S, Baram AB Stachenfeld KL, and Kurth-Nelson Z (2018). What Is a Cognitive Map? Organizing Knowledge for Flexible Behavior. Neuron 100, 490–509. 10.1016/j.neuron.2018.10.002. 30359611

[42] Bottini R, and Doeller CF (2020). Knowledge Across Reference Frames: Cognitive Maps and Image Spaces. Trends Cognit. Sci 24, 606–619. 10.1016/j.tics.2020.05.008. 32586649

[43] Epstein RA, Patai EZ, Julian JB, and Spiers HJ (2017). The cognitive map in humans: spatial navigation and beyond. Nat. Neurosci 20, 1504–1513. 10.1038/nn.4656. 29073650 PMC6028313

[44] Moran R, Dayan P, and Dolan RJ (2021). Human subjects exploit a cognitive map for credit assignment. Proc. Natl. Acad. Sci. USA 118, e2016884118. 10.1073/pnas.2016884118. 33479182 PMC7848688

[45] Jern A, and Kemp C (2015). A decision network account of reasoning about other people’s choices. Cognition 142, 12–38. 10.1016/j.cognition.2015.05.006. 26010559 PMC4500738

[46] Pantelis PC, Baker CL, Cholewiak SA, Sanik K, Weinstein A, Wu C-C, Tenenbaum JB, and Feldman J (2014). Inferring the intentional states of autonomous virtual agents. Cognition 130, 360–379. 10.1016/j.cognition.2013.11.011. 24389312

[47] Barnby JM, Deeley Q, Robinson O, Raihani N, Bell V, and Mehta MA Paranoia, sensitization and social inference: findings from two large-scale, multi-round behavioural experiments. R. Soc. Open Sci 7, 191525. 10.1098/rsos.191525. PMC7137981 32269791

[48] Buhlmann U, Wacker R, and Dziobek I (2015). Inferring other people’s states of mind: Comparison across social anxiety, body dysmorphic, and obsessive-compulsive disorders. J. Anxiety Disord 34, 107–113. 10.1016/j.janxdis.2015.06.003. 26218178

[49] Sripada CS, Angstadt M, Banks S, Nathan PJ, Liberzon I, and Phan KL (2009). Functional neuroimaging of mentalizing during the trust game in social anxiety disorder. Neuroreport 20,984–989. 10.1097/WNR.0b013e32832d0a67. 19521264 PMC2746411

[50] Sharp PB, Dolan RJ, and Eldar E (2023). Disrupted state transition learning as a computational marker of compulsivity. Psychol. Med 53, 2095–2105. 10.1017/S0033291721003846. 37310326 PMC10106291

[51] Seow TXF, Benoit E, Dempsey C, Jennings M, Maxwell A, O’Connell R, and Gillan CM (2021). Model-Based Planning Deficits in Compulsivity Are Linked to Faulty Neural Representations of Task Structure. J. Neurosci 41, 6539–6550. 10.1523/JNEUROSCl.0031-21.2021. 34131033 PMC8318073

[52] Lockwood PL, Apps MAJ, and Chang SWC (2020). Is There a ‘Social’ Brain? Implementations and Algorithms. Trends Cognit. Sci 24, 802–813. 10.1016/j.tics.2020.06.011. 32736965 PMC7501252

[53] Daw ND, Gershman SJ, Seymour B, Dayan P, and Dolan RJ (2011). Model-Based Influences on Humans’ Choices and Striatal Prediction Errors. Neuron 69, 1204–1215. 10.1016/j.neuron.2011.02.027. 21435563 PMC3077926

[54] Vikbladh OM, Meager MR, King J, Blackmon K, Devinsky O, Shohamy D, Burgess N, and Daw ND (2019). Hippocampal Contributions to Model-Based Planning and Spatial Memory. Neuron 102, 683–693.e4. 10.1016/j.neuron.2019.02.014. 30871859 PMC6508991

[55] Kurth-Nelson Z, Economides M, Dolan RJ, and Dayan P (2016). Fast Sequences of Non-spatial State Representations in Humans. Neuron 91, 194–204. 10.1016/j.neuron.2016.05.028. 27321922 PMC4942698

[56] O’Keefe J, and Nadel L (1978). The Hippocampus as a Cognitive Map (Clarendon Press).

[57] Stachenfeld KL, Botvinick MM, and Gershman SJ (2017). The hippocampus as a predictive map. Nat Neurosci advance online publication. 10.1038/nn.4650. 28967910

[58] Constantinescu AO, O’Reilly JX, and Behrens TEJ (2016). Organizing conceptual knowledge in humans with a gridlike code. Science 352, 1464–1468. 10.1126/science.aaf0941. 27313047 PMC5248972

[59] Park SA, Miller DS, and Boorman ED (2021). Inferences on a multidimensional social hierarchy use a grid-like code. Nat. Neurosci 24, 1292–1301. 10.1038/s41593-021-00916-3. 34465915 PMC8759596

[60] Liu Y, Mattar MG, Behrens TEJ, Daw ND, and Dolan RJ (2021). Experience replay is associated with efficient nonlocal learning. Science 372, eabf1357. 10.1126/science.abf1357. 34016753 PMC7610948

[61] Doll BB, Duncan KD, Simon DA, Shohamy D, and Daw ND (2015). Model-based choices involve prospective neural activity. Nat. Neurosci 18, 767–772. 10.1038/nn.3981. 25799041 PMC4414826

[62] Palan S, and Schitter C (2018). Prolific.ac–A subject pool for online experiments. Journal of Behavioral and Experimental Finance 17, 22–17. 10.1016/j.jbef.2017.12.004.

[63] Salvatier J, Wiecki TV, and Fonnesbeck C (2016). Probabilistic programming in Python using PyMC3. PeerJ Comput. Sci 2, e55. 10.7717/peerj-cs.55. PMC10495961 37705656

[64] Zhou Z, Bloem M, and Bambos N (2018). Infinite Time Horizon Maximum Causal Entropy Inverse Reinforcement Learning. IEEE Trans. Automat. Control 63, 2787–2802. 10.1109/TAC.2017.2775960

[65] Russek EM, Momennejad I, Botvinick MM, Gershman SJ, and Daw ND (2017). Predictive representations can link model-based reinforcement learning to model-free mechanisms. PLoS Comput. Biol 13, e1005768. 10.1371/journal.pcbi.1005768. 28945743 PMC5628940

[66] Hoffman MD, and Gelman A (2014). The No-U-Turn Sampler: Adaptively Setting Path Lengths in Hamiltonian Monte Carlo. J. Mach. Learn. Res 15, 1593–1623.

[67] Phan D, Pradhan N, and Jankowiak M (2019). Composable effects for flexible and accelerated probabilistic programming in NumPyro. Preprint at arXiv, 1912.11554. 10.48550/arXiv.1912.11554.

[68] Kocsis L, and Szepesvári C (2006). Bandit Based Monte-Carlo Planning In Machine Learning: ECML 2006 Lecture Notes in Computer Science, Fürnkranz J, Scheffer T, and Spiliopoulou M, eds. (Springer), pp. 282–293. 10.1007/11871842_29.

[69] Gelly S, and Silver D (2007). Combining online and offline knowledge in UCT. In Proceedings of the 24th International Conference on Machine Learning ICML ‘07 (Association for Computing Machinery)), pp. 273–280. 10.1145/1273496.1273531.

[70] Finnsson H, and Björnsson Y (2008). Simulation-based approach to general game playing. In Proceedings of the 23rd National Conference on Artificial Intelligence - Volume 1 AAAI’08 (AAAI Press)), pp. 259–264.

[71] Cranmer K, Brehmer J, and Louppe G (2020). The frontier of simulation-based inference. Proc. Natl. Acad. Sci. USA 117, 30055–30062. 10.1073/pnas.1912789117. 32471948 PMC7720103

[72] Greenberg DS, Nonnenmacher M, and Macke JH (2019). Automatic posterior transformation for likelihood-free inference. Preprint at arXiv. 10.48550/arXiv.1905.07488.

