# Supplementary material for: Interactive cognitive maps support flexible behavior under threat
Source: Cell Rep. Author manuscript; Available in PMC 2023 Nov 20. (PMC10658881; doi:10.1016/j.celrep.2023.113008)
Supplement: 1 [file NIHMS1928300-supplement-1.pdf]

Cell Reports, Volume *42*

Supplemental information

Interactive cognitive maps support flexible behavior under threat

Toby Wise, Caroline J. Charpentier, Peter Dayan, and Dean Mobbs

Interactive cognitive maps support flexible behavior under threat

Toby Wise[1,2*], Caroline J Charpentier[2,3,4], Peter Dayan[5,6] & Dean Mobbs[2,7]

[1]Department of Neuroimaging, Institute of Psychiatry, Psychology & Neuroscience, King's College London, London, United Kingdom

[2]Department of Humanities and Social Sciences and California Institute of Technology, Pasadena, California, USA.

[3]Department of Psychology, University of Maryland, College Park, Maryland, USA

[4]Brain and Behavior Institute, University of Maryland, College Park, Maryland, USA

[5]Max Planck Institute for Biological Cybernetics, Tübingen, Germany

[6]University of Tübingen, Tübingen Germany

[7]Computation and Neural Systems Program at the California Institute of Technology, California, USA

*Lead contact (toby.wise@kcl.ac.uk)

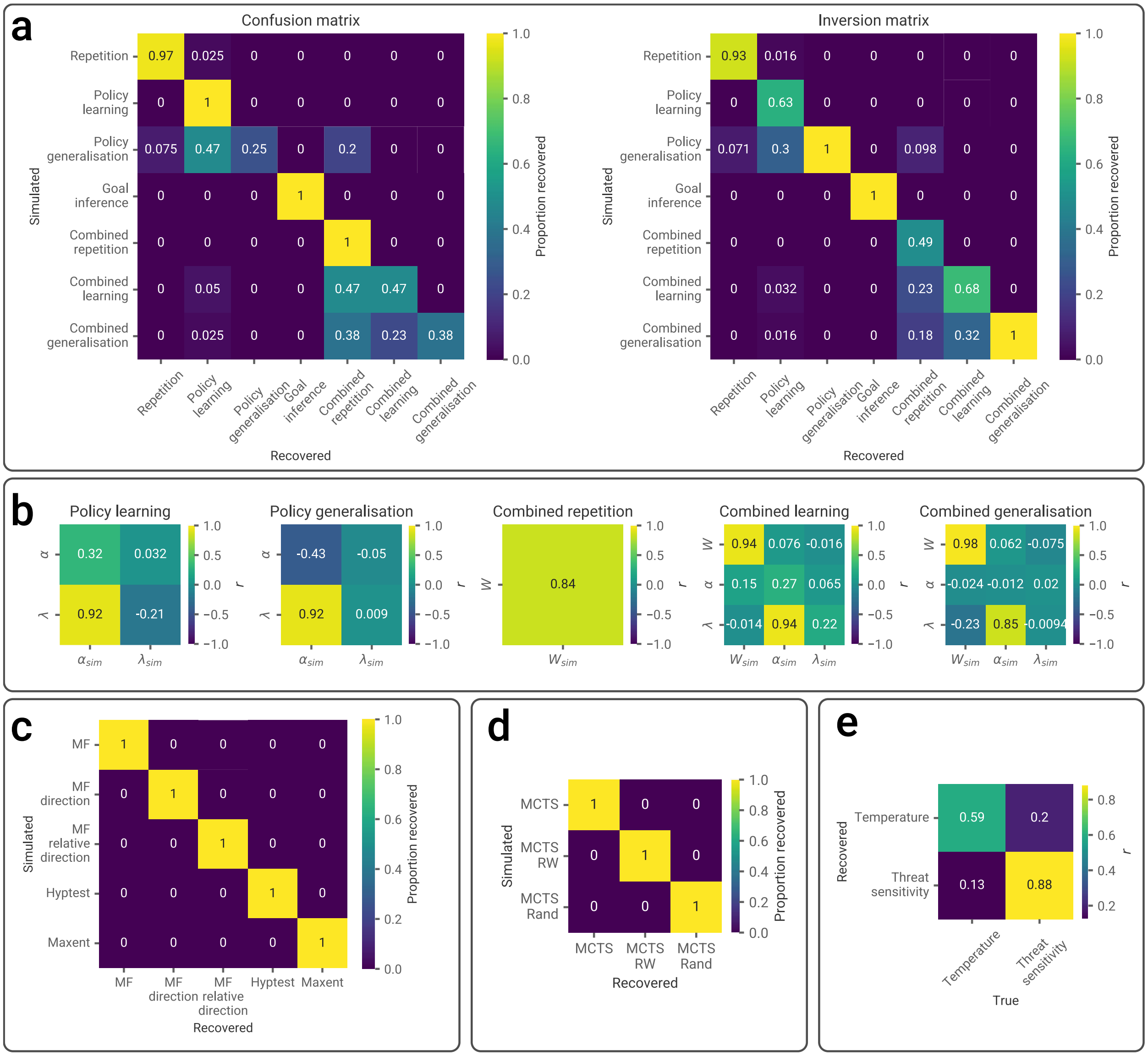

Figure S1. Model and parameter recovery analyses. A) Model recovery for the action prediction models. The right panel shows the confusion matrix (representing $p(\text{recovered model} \mid \text{simulated model})$) while the left shows the inversion matrix ($p(\text{simulated model} \mid \text{recovered model})$). While there was some confusion between the model free approaches, the critical distinction between these and approaches based on goal inference was recovered with near perfect accuracy. B) Parameter recovery for the action prediction models. Values represent Pearson r correlation coefficients. When assessing parameter recovery within these models, we found that the weighting parameter, representing the balance between model-free and goal inference approaches, was recovered accurately across all models (minimum correlation between true and estimated values = .84. In contrast, learning rates and learning rate decay parameters were not recovered accurately, with high decay rates often resulting in a high recovered learning rate rather than a high recovered decay rate. However, these parameters were not central to any of our analyses. W = weighting parameter, α = learning rate, λ = learning rate decay. C) Model recovery for the inverse reinforcement learning models. MF = model free, MaxEnt = Maximum Entropy, HypTest = Hypothesis Testing. D) Model recovery for the MCTS planning models. MCTS = model not accounting for predator, MCTS rand = model assuming predator behaves randomly, MCTS RW = model assuming predator acts in a goal-directed manner based on its reward weights. E) Parameter recovery for the softmax temperature and threat sensitivity parameters in the MCTS planning model.

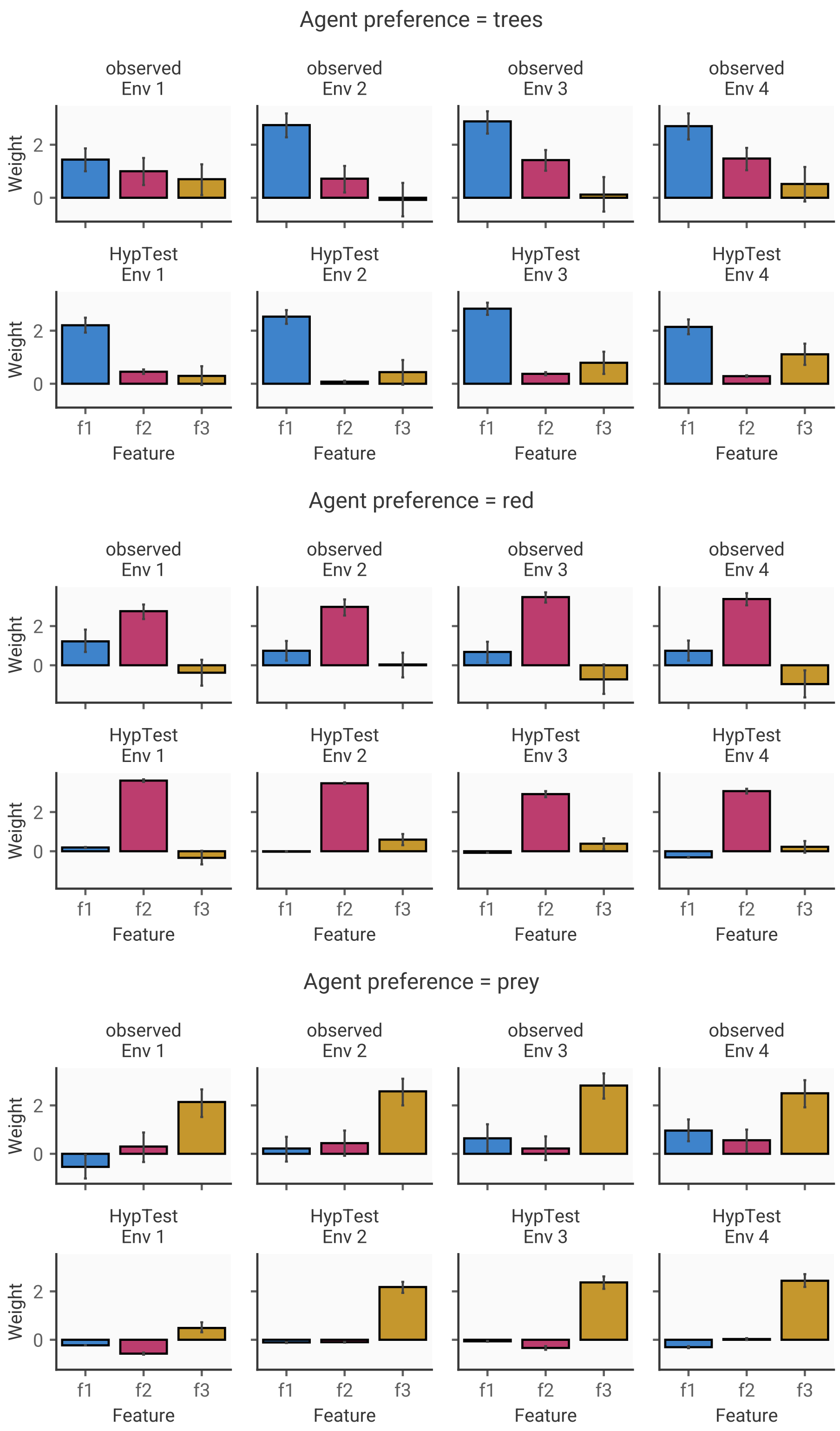

Figure S2. Hypothesis testing inverse reinforcement learning model predictions for each environment in Experiment 1, across each of the three preference conditions. Observed = participant responses, HypTest = model predictions.
